# Optical Spectral Fingerprinting Enables Sensitive Detection of Anthracycline Chemotherapeutics in Synthetic Clinical Biofluids

**DOI:** 10.1021/acs.nanolett.6c01777

**Published:** 2026-07-06

**Authors:** Atara R. Israel, Yunjung Kim, Adnan Arnaout, Myesha Thahsin, Yumna Ahmed, Zachary Cohen, Amelia Ryan, Syeda Rahman, Mijin Kim, Ryan M. Williams

**Affiliations:** ‡ Department of Biomedical Engineering, 14770 The City College of New York, New York, New York 10031, United States; § School of Chemistry and Biochemistry, 1372 Georgia Institute of Technology, Atlanta, Georgia 30332, United States; ⊥ Department of Chemistry, 26716 Hanyang University, Seoul 04763, Republic of Korea; ∥ Department of Medicine, Division of Nephrology and Hypertension, Stony Brook University, Stony Brook, New York 11794, United States

**Keywords:** doxorubicin, single-walled carbon nanotubes, SWCNT, near-infrared (NIR) fluorescence, cardiotoxicity, machine learning

## Abstract

Anthracycline chemotherapeutics are common chemotherapeutics that have substantial toxicities. There is substantial interpatient pharmacokinetic variability, though there is no method to quantify organ or tumor exposure. Here, we exposed an optical nanosensor array to detect each of four anthracyclines. We screened 12 ssDNA sequences paired with seven single-walled carbon nanotube (*n*,*m*) species against several concentrations of doxorubicin, daunorubicin, idarubicin, and epirubicin. Complex spectral responses were used to develop machine-learning-based classification models to quantify each anthracycline. The optimized extreme gradient boosting model classified high levels of each anthracycline with 100% accuracy. Principal component analysis distinguished low (≤5 μM) and high concentrations of each anthracycline. Finally, we validated selected ssDNA-(*n*,*m*) pair performance in synthetic urine and sweat. Our findings deliver a generalizable optical spectral fingerprinting methodology for hard-to-detect analytes. Their use in clinical biofluids portends the preclinical and potentially clinical pharmacokinetic measurement of anthracyclines to improve efficacy and reduce toxicities.

## Graphical abstract

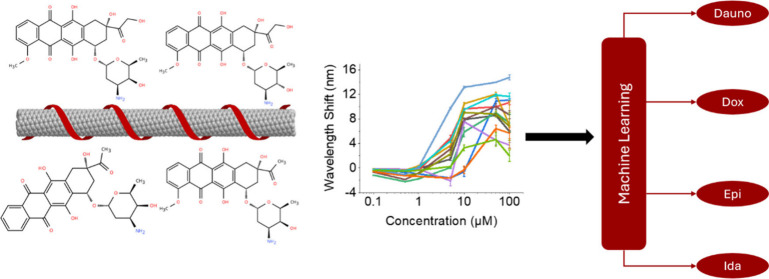

Anthracyclines are a potent class of chemotherapeutics derived from bacteria and widely used in the treatment of breast cancer, certain sarcomas, and leukemia/lymphomas.^1^ Anthracyclines exhibit toxicity to both cancerous and healthy cells in several ways, including DNA intercalation, topoisomerase IIβ inhibition,^2,3^ and cardiolipin antagonism.^4^ Despite their efficacy, clinical anthracycline use is limited due to the lifetime dose-dependent cardiotoxicity and other side effects.^2,5^ These adverse effects can manifest both acutely and as long-term sequelae.^6^ Current methods used to diagnose cardiac function include evaluation of left ventricle ejection fraction by echocardiography, or cardiac biopsy.^7^ Despite established guidelines,^8^ anthracycline dosage is subject to substantial interpatient variability with a narrow therapeutic window. Thus, precise pharmacokinetic monitoring would be beneficial to optimize therapeutic outcomes while minimizing serious adverse events. Preclinical and clinical development methods for measuring anthracycline exposure in the body include mass spectrometry,^9^ high-performance liquid chromatography,^10^ and the quantification of biomarkers of anthracycline-induced cardiotoxicity.^7^ These methods, however, are expensive and invasive and/or may be too late for impact.

Semiconducting single-walled carbon nanotubes (SWCNTs) are quasi-one-dimensional nanomaterials comprised of sp^2^-hybridized carbon atoms. SWCNTs emit stable near-infrared (NIR) fluorescence,^11^ that is sensitive to changes in the local environment.^12^ Many SWCNT chiralities exist, each defined by a distinct (*n*,*m*) index that specifies the nanotube diameter and chiral angle, each with individual absorbance and optical emission maxima.^13^ The unique structure and electronic properties of (*n*,*m*) SWCNTs enable different interactions with their local environment.

Prior studies have used SWCNTs as transducers in sensors for a variety of clinically relevant analytes, including lipids,^14^ small molecule neurotransmitters,^15–17^ glutathione-*S*-transferase (GST) fusion proteins,^18^ riboflavin,^19,20^ and other chemotherapeutic agents such as cisplatin and transplatin.^21^ SWCNT sensors have also been utilized to detect several biomarkers in patient samples, including estrogen receptor α^22^ and several gynecological markers.^23,24^ Implantable devices containing SWCNT have been engineered for in vivo sensor deployment to detect cancer markers,^25^ steroid hormones^26^ and others.^27^ Among chemotherapeutics, nanotube-based doxorubicin detection has been well investigated.^28–30^ However, these SWCNTs function as generic anthracycline sensors rather than being specific to a single type.^28–30^

To improve the specificity of anthracycline sensing, we employed the spectral fingerprinting method.^24,31,32^ Spectral fingerprinting extends the molecular perception concept by correlating fluorescent responses from the sensor array with disease states, underlying cellular processes, or mixtures of analytes, and incorporates machine-learning methods to discern these correlations. Machine-learning-based data analyses approaches have also been used to understand SWCNT photophysical properties^33^ and their interactions with analytes.^34^ In this work, we developed a SWCNT fluorescence spectral fingerprinting method to detect four structurally similar anthracyclines—doxorubicin, daunorubicin, epirubicin, and idarubicin (Figure 1). The array consisted of 12 ssDNA sequences, some of which had been used for anthracycline detection and variants thereof, while we analyzed fluorescence intensity and wavelength changes from seven (*n*,*m*) species. We exposed the array to seven concentrations of each of the four anthracyclines in buffer, synthetic sweat, and synthetic urine. Fluorescence responses of ssDNA-SWCNTs were analyzed to differentiate among four anthracyclines, with the machine-learning model achieving 100% classification accuracy. Quantitative sensitivity to each anthracycline, assessed using both traditional binding kinetic fits and machine-learning models, achieved up to 100% accuracy in classifying concentration-dependent responses to daunorubicin and idarubicin. This work establishes the use of optical nanotube-based sensors that exhibit distinct responses to two anthracyclines, along with machine-learning models to distinguish between different anthracyclines and to predict concentration.

**Figure 1:**
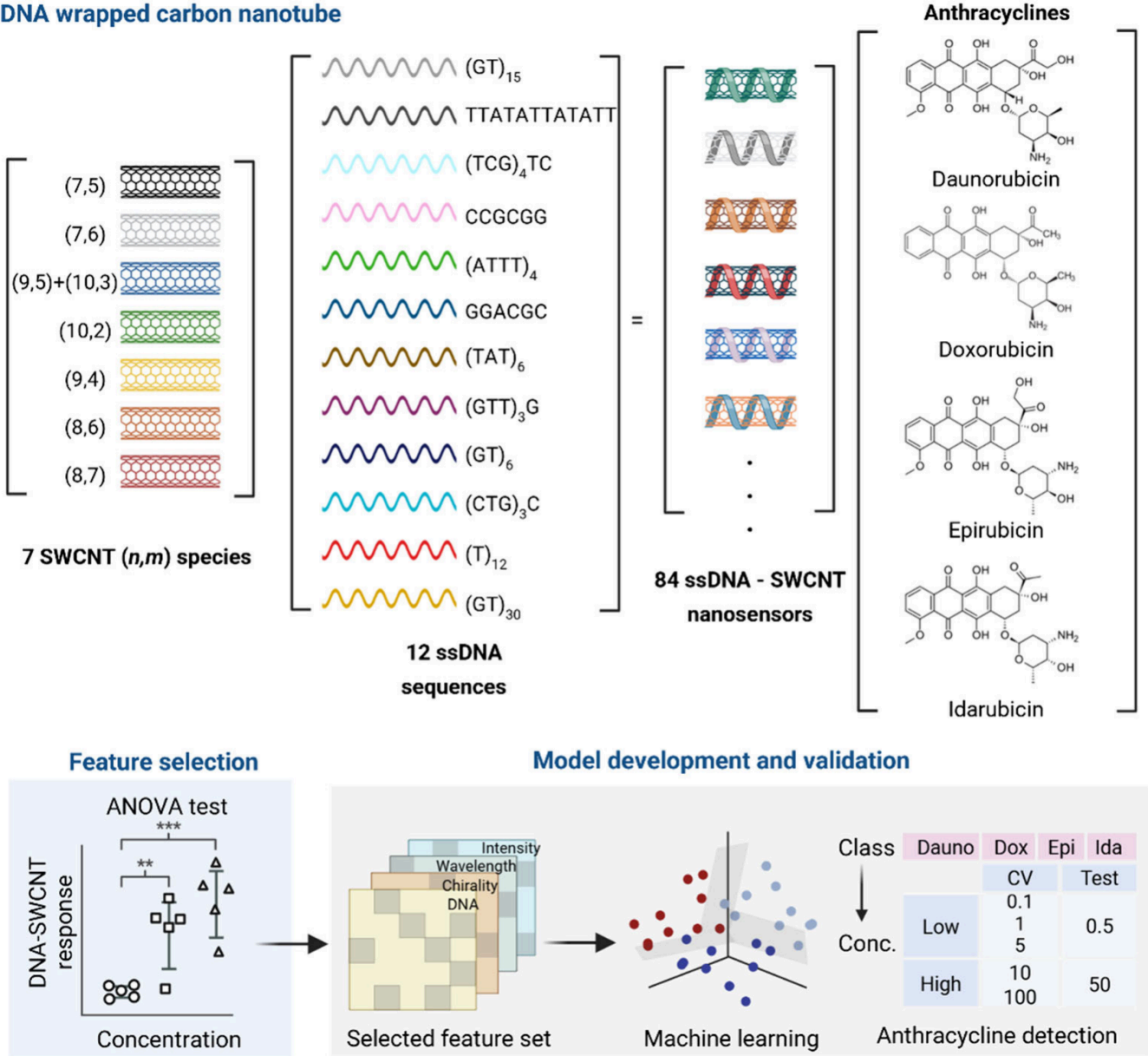
Workflow of nanosensor array screening and machine learning for anthracycline detection. Top: Dispersion of mixed-species SWCNT with ssDNA sequences, followed by the addition of anthracyclines to each sensing platform. Bottom: Machine learning to determine optimal anthracycline sensors for classification based on anthracycline class and concentration.

## Construction and Characterization of a SWCNT Sensor Array

SWCNTs were first encapsulated with 12 different ssDNA sequences, with subsequent analysis of 7 (*n*,*m*) structures to create 84 unique sensing elements (Figure 1). ssDNA sequences were selected from SWCNT for species sorting and molecular sensing as previously reported. (GT)_15_ has been employed for anthracycline detection^28,30^ and other small molecule detection,^16,20,24,35,36^ along with (GT)_6_,^31,35,37,38^ and (GT)_30_.^39^ Others, including TTATATTATATT,^40,41^ GGACGC,^42^ (TCG)_4_TC,^43^ (GTT)_3_G,^43,44^ and (ATTT)_4_,^45^ have been used in chiral separation techniques, as some nucleotide sequences create unique conformations with SWCNT. Variations of previously reported poly-CG sequences, CCGCGG and (CTG)_3_C, were also included.^42,43,46^ (TAT)_6_ was previously used for dispersion prior to antibody conjugation^22,25,45,47^ and is a variant of several poly-TA sequences for chirality sorting.^43,44^ (T)_12_ was included because it forms a somewhat weaker left-handed helical wrapping on SWCNT, allowing for more rearrangement.^48^

DNA-SWCNT complexes were characterized by UV–vis absorption spectroscopy, followed by NIR fluorescence spectroscopy, using excitation wavelengths of 655 and 730 nm (Supplementary Figure 1). Absorbance spectra demonstrated stable SWCNT suspensions. Some sequences, for example, (ATTT)_4_ and GGACGC, resulted in stronger dispersion and higher absorbance than others, such as CCGCGG. All DNA-SWCNT constructs produced bright emission peaks from several (*n*,*m*) species. Variance in brightness of (*n*,*m*) species demonstrated heterogeneity imparted by differential sequence interaction. As an example, (ATTT)_4_-SWCNT exhibited minimal fluorescence from the (8,3) and (9,5) + (10,3) species when excited at 655 nm. The (TAT)_6_-wrapped (7,6) SWCNT was dimmer, while the (8,7) species was brighter compared to other sequences evaluated. The (GT)_6_-wrapped (10,2) SWCNT were much brighter in comparison to all other ssDNA-SWCNT combinations. Predominant across all constructs evaluated, the (7,5), (7,6), and (9,4) species were the brightest at the excitation wavelengths used.

## Anthracyclines Induce Concentration-Dependent Spectral Responses of the Nanosensor Array

SWCNT suspensions were exposed to doxorubicin, daunorubicin, epirubicin, and idarubicin at concentrations of 100, 50, 10, 5, 1, 0.5, and 0.1 μM (Figure 2 and Supplementary Figures 2–7). We observed 20–40 nm redshifts for a majority of ssDNA-SWCNT species assessed when incubated with 100 μM anthracycline, similar to our previous findings that studied the effect of doxorubicin on (GT)_15_-SWCNTs.^28,30^ Smaller magnitude redshifts, or slight blueshifts, were observed as the anthracycline concentration decreased. In contrast, intensity changes did not follow typical dose–response kinetics (Figure 2A–D and Supplementary Figures 2–7). While most (*n*,*m*) exhibited fluorescence quenching up to 80% in the presence of high anthracycline concentrations, (TAT)_6_-SWCNT brightness increased in response to daunorubicin and idarubicin across all (*n*,*m*) species. This result is similar to a previous report of a SWCNT doxorubicin sensor dispersed with the ssDNA sequence C_8_AGA_2_T_2_ACT_2_C_8_ (referred to as CB-13), which also observed brightening intensity and redshifting of up to 17 nm of the (7,6) species.^29^ Other ssDNA-SWCNT combinations in that study, however, observed a trend of quenched intensity and up to 8 nm redshifts in response to doxorubicin. Our work indeed had overlapping sequence space with that study, notably (GT)_15_ and (ATTT)/(TAT) repeats, in which we found similar responses to doxorubicin. As that study did not investigate other anthracyclines, the divergence of responses for (TAT) but not (ATTT) repeats are notable. Interestingly, that study found a weak correlation between the ability of a sequence to suspend SWCNT and doxorubicin response.^29^ Future studies may integrate both the library assembled here and the poly-C-flanked library from that work to enhance sequence space. In total, the divergence of responses is driven by the unique conformation each ssDNA sequence adopts in relation to the chiral angle and diameter of diverse (*n*,*m*) species.^48,49^ Uncoated SWCNT surface area allows direct analyte interaction.^41^ Changes in fluorescence result from charge transfer and/or polarity modulation from the anthracycline,^50^ with additional contributions from ssDNA restructuring.^37,48,51^

**Figure 2:**
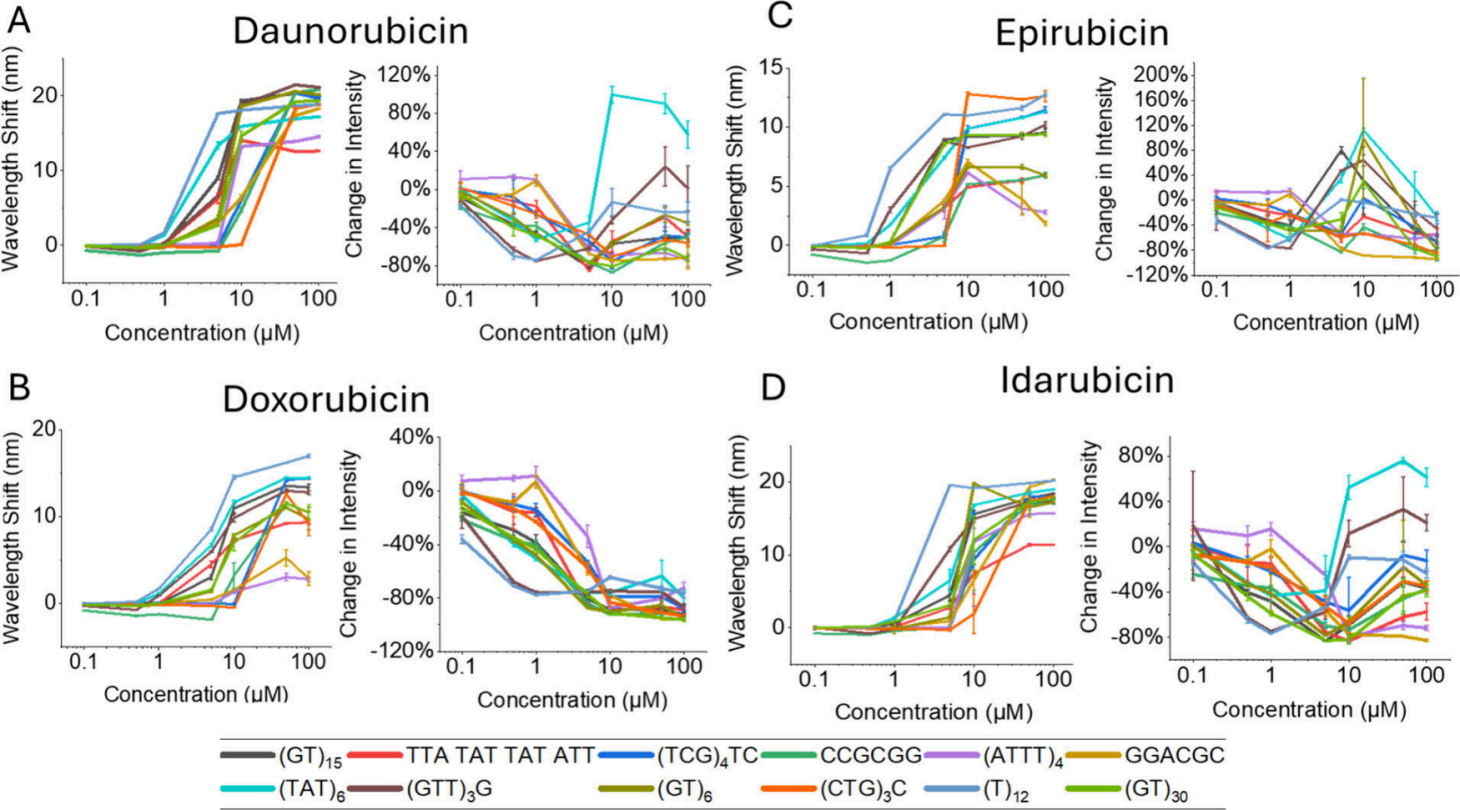
Concentration curves demonstrating shifts in the center wavelength and maximum intensity of (7,5)-SWCNTs: (A) daunorubicin; (B) doxorubicin; (C) epirubicin; (D) idarubicin.

Our prior work demonstrated that doxorubicin binding does not displace ssDNA on the nanotube surface.^28^ The aromatic structure of anthracyclines, however, does have a strong affinity for the nanotube surface.^52^ The affinity enables both direct charge transfer and polarity modulation. Anthracyclines are also strong DNA intercalators,^53^ altering the ssDNA conformation on the nanotube surface and thereby fluorescence emission. This is supported by other studies which synthesized SWCNT-based electrochemical sensors with dsDNA, finding a reduction in voltametric peaks upon addition of anthracyclines, attributed to intercalation of the drugs with DNA.^54,55^ Several other optically active nanomaterials, such as quantum dots and metal nanoclusters, have been designed for anthracycline detection, with the primary detection method being quenched absorbance or fluorescence. This is similar to our observations of SWCNT-anthracycline interactions here. The use of nanomaterials as a means to detect anthracyclines is a strong alternative to traditional methods due to the high affinity enabled by the surface chemistry of nanomaterials, and the lack of recognition elements for this drug class.^56^

We found that the theoretical limits of detection (LOD; 3*σ_control_) ranged between 0.017 and 7.4 μM for wavelength-dependent modulations and between 0.1 and 5 μM for intensity-dependent modulations across ssDNA sequences and (*n*,*m*) species (Supplementary Table 1). The lowest wavelength-dependent LOD was obtained by (TAT)_6_-(7,5), while the lowest intensity-dependent LOD was obtained by (TCG)_4_TC-(8,7). Interestingly, the highest LODs were observed in the (8,7) and (9,5) + (10,3) peaks, suggesting a trend of higher LOD for larger-diameter species. Generally, we observed large wavelength shifts or intensity modulations in response to 10–100 μM anthracycline. At 5 μM, we observed notable variability: whereas some sensor combinations exhibited fluorescence modulations, others demonstrated almost no change. This is also true, to a lesser extent, at 1 μM. The addition of 0.5 and 0.1 μM anthracycline induced no substantial changes in fluorescence output. Other nanosensors for doxorubicin have found lower LOD, in some cases subnanomolar.^56^ To cover a broad range of anthracycline concentrations with high accuracy, it is necessary to include multiple sensor constructs in a single model.

## Anthracycline-SWCNT Binding Kinetics

We next evaluated binding kinetics for individual ssDNA-(*n*,*m*) combinations using a standard noncooperative binding model^30^ (Figure 3 and Supplementary Table 2). Dissociation constants (*K*_d_) in the submicromolar range indicated strong binding affinities of most ssDNA-(*n*,*m*) constructs for anthracyclines, which is supported by previous studies.^28^ We used *K*_d_ values to identify top-performing ssDNA-(*n*,*m*) pairings for each anthracycline (Supplementary Table 2). For daunorubicin, intensity modulations of GGACGC-(8,7) exhibited the strongest binding, with a *K*_d_ of 0.18 μM. Doxorubicin binding to (ATTT)_4_-(10,2) exhibited a *K*_d_ of 0.13 μM. We observed optimal epirubicin detection with (CTG)_3_C-(9,4), with an intensity change *K*_d_ of 0.26 μM, while wavelength shifts in CCGCGG-(8,7) were optimal for idarubicin, with a *K*_d_ of 0.62 μM. In general, average dissociation constants for all anthracyclines were relatively similar, with the average *K*_d_ for daunorubicin being 7.6 μM, doxorubicin being 8.1 μM, and epirubicin and idarubicin being 7.0 and 5.8 μM, respectively. The range of *K*_d_ values, however, was large. For daunorubicin, *K*_d_ values ranged from 0.18 to 60.3 μM, while for doxorubicin and epirubicin, wider *K*_d_ ranges of 0.13–100.1 and 0.26–194.9 μM were seen. Idarubicin responses exhibited a range of *K*_d_ values between 0.62 and 74.7 μM. The strong π–π stacking, aromatic binding, and electrostatic interactions between SWCNT and anthracyclines is supported by these relatively strong binding affinities.^28,57,58^ Prior (GT)_15_-SWCNT doxorubicin detection exhibited generally lower binding affinities quantified by intensity changes than those in our study.^30^ We also found a wider range of dissociation constants than did prior work with a turn-on doxorubicin-SWCNT sensor.^29^

**Figure 3:**
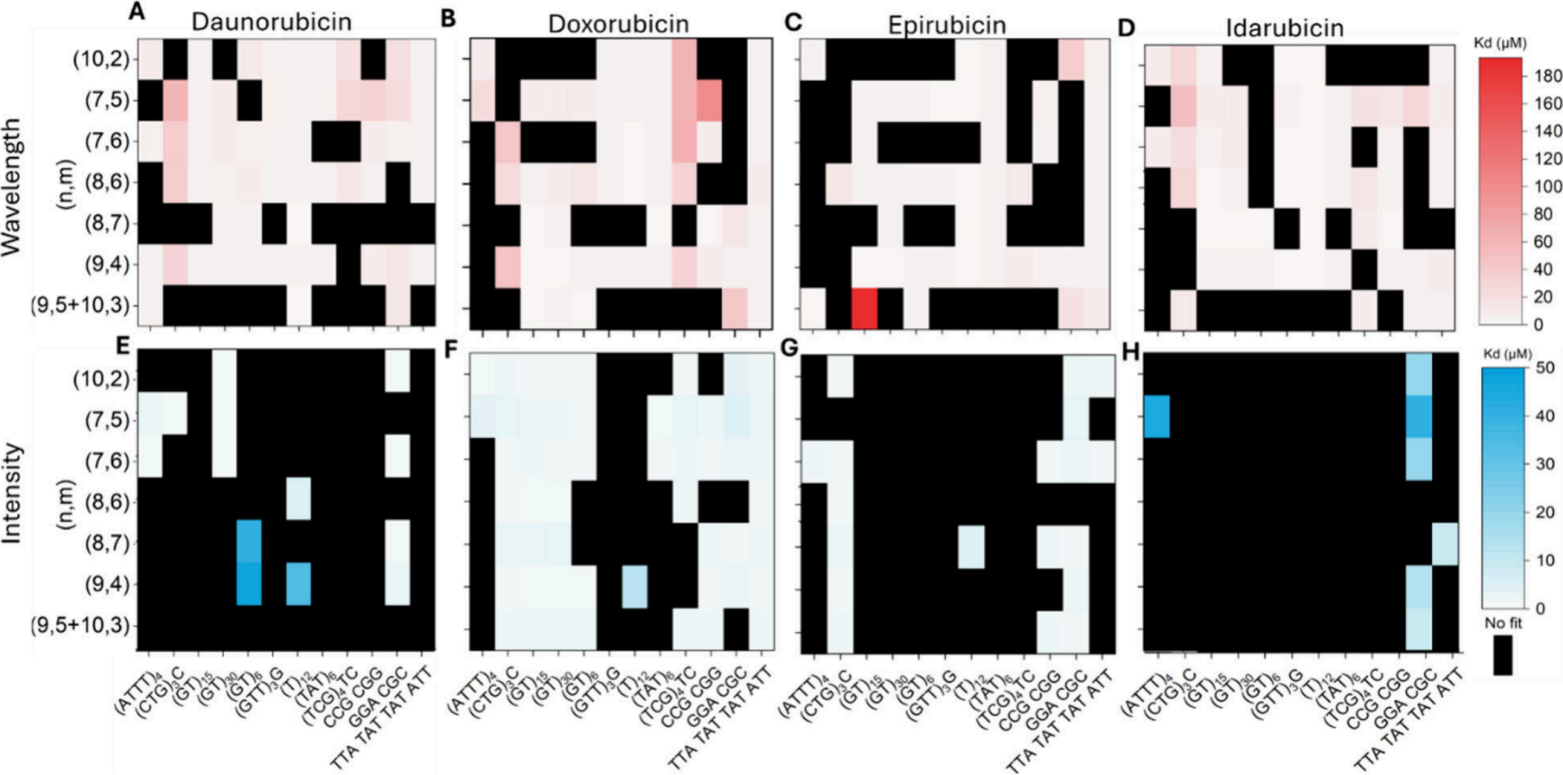
Dissociation constant of each ssDNA-(*n*,*m*) construct. The Hill equation was used to fit binding kinetics and obtain a dissociation constant (*K*_d_) for all SWCNT constructs for both wavelength and intensity-induced fluorescence changes in the presence of daunorubicin (A and E), doxorubicin (B and F), epirubicin (C and G), and idarubicin (D and H). Black = no fit.

## Statistical Assessment of Significant Sensor Responses

Given different outcomes in optimal ssDNA-(*n*,*m*) species for LOD and dissociation constant, we next sought to determine which specific features of fluorescence response data best predict the presence of an anthracycline. As the screening experiments yielded 14,112 data points, we used machine-learning models to identify spectral fingerprints that improve sensing for each anthracycline (Figure 1). First, we ranked data set features, comprising ssDNA-(*n*,*m*) combinations and their corresponding spectral responses, using ANOVA. Spectral responses significantly different than controls (*p* < 0.05) at two or more concentration levels for at least one type of anthracycline were included in the feature set. This reduced the feature dimensionality by 57%, yielding 48 statistically significant ssDNA-(*n*,*m*) combinations with 93 spectral features. The 93 spectral features comprised 45 ssDNA-(*n*,*m*) constructs exhibiting concurrent changes in both wavelength and intensity, and three species characterized by changes in either wavelength or intensity.

The selected nanosensors exhibited diverse response behaviors, with some showing monotonic intensity changes and others displaying wavelength shifts, reflecting heterogeneous ssDNA-(*n*,*m*) interactions with different target analytes. Interestingly, both wavelength shift and intensity changes demonstrated similar patterns in fluorescence modulations for ssDNA-(*n*,*m*) pairings, but the magnitude of change of the spectral signatures was slightly different (Figure 4). The most significant features were observed with (CTG)_3_C, which showed 24 significant features for anthracycline and spectral feature pairings. This was followed by TTATATTATATT, (TCG)_4_TC, and (GTT)_3_G, which all had 16–18 significant features. Each of these sequences are 10–14 bases, which suggests there may be an optimal length for anthracycline detection. Interestingly, however, (GT)_15_-SWCNT had more significant features among poly­(GT) sequences. Among SWCNT species, <0.9-nm-diameter (7,5), (7,6), and (9,4) SWCNTs were identified as high-contributing features in the ANOVA-based determination of the number of significant spectral responses observed due to anthracycline perturbations, with 32–39 significant features each. Fewer significant features were observed in the (8,7), (8,6), and (9,5) + (10,3) peaks, all of which are >0.95 nm in diameter. Contradictorily, the <0.9-nm-diameter (10,2) also demonstrated fewer significant features than the others of this diameter regime. Prior work has demonstrated that variability in certain ssDNA wrappings may facilitate greater responsiveness among certain (*n*,*m*).^44^ Indeed, in some cases, the smaller-diameter species have been more responsive compared to larger-diameter species.^59^ While these results suggest some diameter dependence, the confounding nature of the (10,2) response indicates that SWCNT responsivity is likely driven by other factors as well, including the possibility of spectral congestion from closely emitting species.^60^

**Figure 4:**
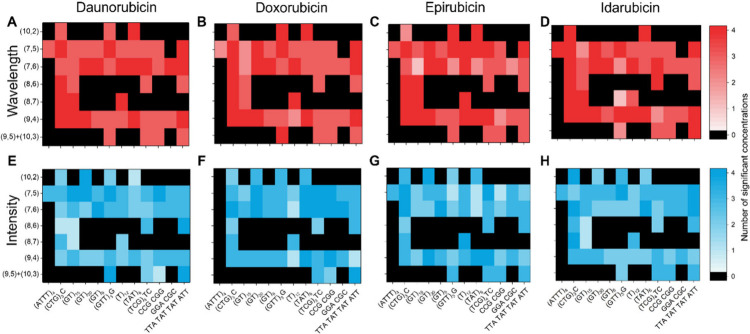
Heatmap plots by the ANOVA test for statistically significant feature selection. Wavelength (A–D) and intensity (E–H) changes of each ssDNA (row)–(*n*,*m*) (column) combination were correlated with the four concentrations (0.1, 1, 10, and 100 μM) of daunorubicin (A and E), doxorubicin (B and F), epirubicin (C and G), and idarubicin (D and H). Legend scale 1–4 indicates the number of concentrations at which each combination showed a significant difference, with black indicating none.

## Multiclass Classification of Anthracyclines

The selected feature set of 48 nanosensors was subsequently used as input for principal componenet analysis (PCA). The PCA plot showed the variance in spectral responses across the separate anthracycline classes. While the score plot of the first two principal components (PC1 and PC2) collectively explained 77% of the total variance, the discrimination between anthracycline types was limited, leaving substantial overlap among the four analytes (Supplementary Figure 8). This poor separability in PCA indicates the limitations of unsupervised linear dimensionality reduction for anthracycline discrimination, suggesting that more sophisticated machine-learning models are needed.

To evaluate classification performance, three machine-learning models were implemented: decision tree (DT), support vector machine (SVM), and eXtreme Gradient Boosting (XGBoost). The models were trained on spectral responses at 0.1, 1, 10, and 100 μM, and their performance was evaluated on an independent test set with intermediate concentrations of 0.5, 5, and 50 μM. Using 3-fold cross-validation, the models achieved accuracies of 94 ± 12% (DT), 98 ± 4% (SVM), and 100% (XGBoost). Test set performance confirmed these results with accuracies of 100%, 50%, and 100%, respectively (Figure 5A).

**Figure 5:**
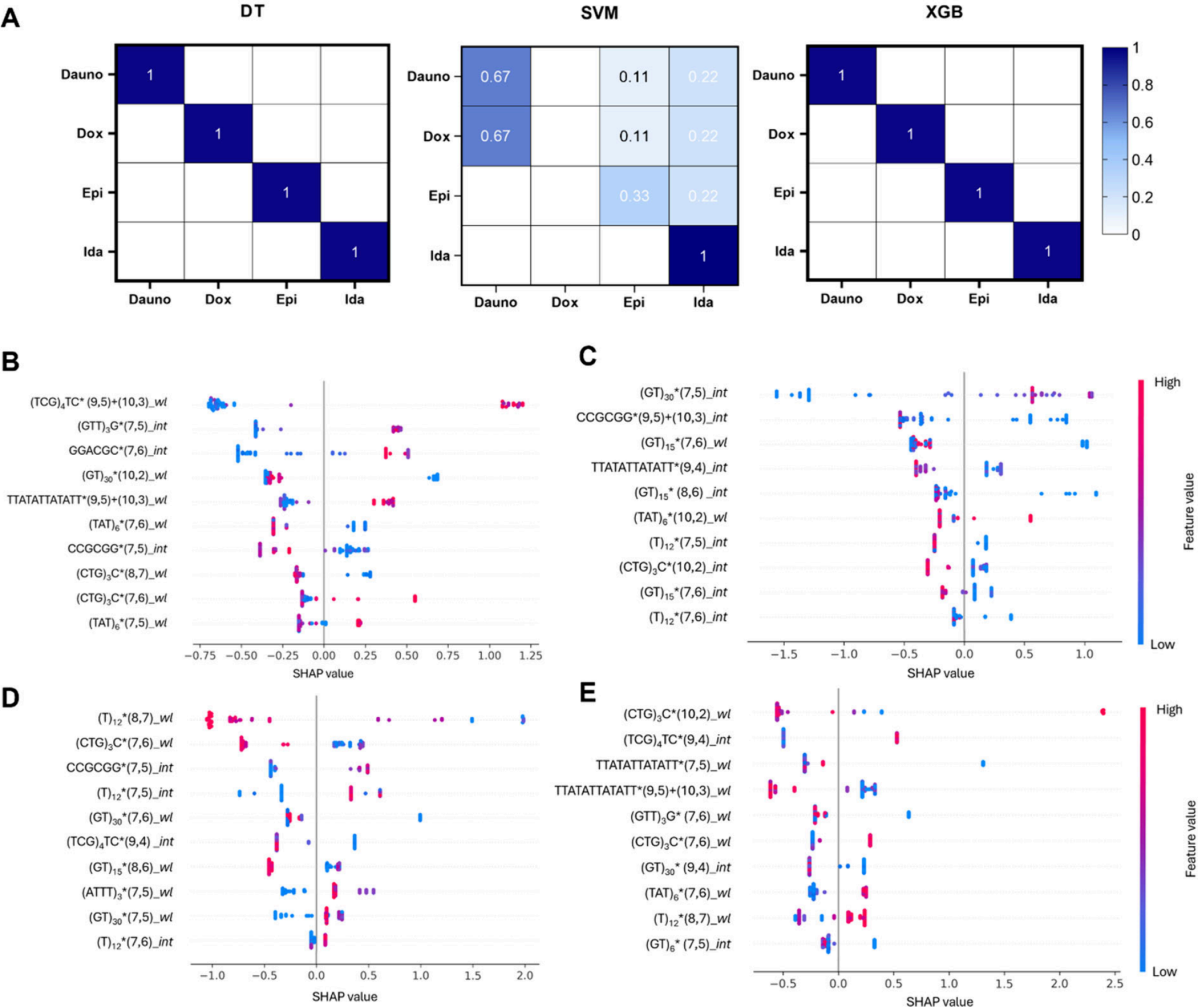
Machine learning to determine optimal features for anthracycline detection. (A) Confusion matrix of the three machine-learning models for discriminating four anthracycline classes in the independent test set. SHAP values indicating feature importance for each anthracycline discrimination against others: (B) daunorubicin; (C) doxorubicin; (D) epirubicin; (E) idarubicin. The colors on the symbols indicate the magnitudes of corresponding input variables: red and blue are for higher and lower values, respectively.

To define the most discriminable features for anthracycline classification, we calculated SHAP (SHapley Additive exPlanations) values in the optimized XGB model (Figure 5B–E). By integrating local feature attributions into a unified global interpretability framework, SHAP analysis provided insight into which spectral characteristics most strongly influenced the decision boundaries of the models. Consequently, the derived SHAP values served as an effective means to associate the machine-learning predictions with underlying physicochemical variations among the anthracycline molecules. SHAP values for each anthracycline revealed the contribution of each feature to predicting anthracycline type. Features exhibiting the largest absolute SHAP values, such as wavelength shifts for (TCG)_4_TC-(9,5)+(10,3) and intensity changes of (GTT)_3_G-(7,5), demonstrated the strongest contributing features for distinguishing daunorubicin from other anthracyclines. For doxorubicin, (GT)_30_-(7,5) intensity change and CCGCGG-(9,5)+(10,3) intensity change had the highest SHAP values. The highest-ranking sensor constructs for epirubicin were shifts in (T)_12_-(8,7) and (CTG)_3_C-(7,6). For idarubicin sensing, the top-ranked sensor constructs were wavelength shifts in (CTG)_3_C-(10,2) and intensity change in (TCG)_4_TC-(9,4). These compound-specific classifying features demonstrated that the detection of each anthracycline is characterized by a unique subset of highly informative ssDNA-(*n*,*m*) combinations that reflect differential binding affinities and sensitivities. It is well-known that aromatic compounds interacting with the SWCNT surface allow for π–π stacking.^57,61^ In addition, the intercalation of anthracyclines with DNA is also well-characterized.^62^ Previous molecular dynamics simulations found that DNA sequences and doxorubicin or idarubicin found they held the strongest bonds with cytosine and guanine rings,^55^ which are present in several of the SHAP top-selected sequences. Notably, several of the lowest-selected sequences contained only thymine or adenine, though not all. Another study also found that doxorubicin induced redshifting and a greater intensity change in SWCNT with longer emission wavelength,^29^ which correlates with the selected SHAP pairings for daunorubicin, doxorubicin, and epirubicin. These observations are somewhat opposed to prior observations regarding small-diameter SWCNT demonstrating more significant features (Figure 4); however, it is good evidence that the machine-learning models weight contributors to sensing differently.

## Concentration-Based Binary Classification

We next investigated whether the sensor array could discriminate variations in anthracycline concentration. First, PCA was performed to visualize concentration-dependent clustering patterns for each anthracycline. By transforming the 93 spectral features into a reduced set of principal components (PCs), major chemical variations were captured within a lower-dimensional space. To further evaluate concentration discrimination capability, PCA was applied to samples for five concentrations (0.1, 1, 5, 10, and 100 μM) to establish the principal component space. The score plots of the first two principal components demonstrate general clustering of low and high concentrations for each anthracycline (Figure 6). Subsequently, test samples at intermediate concentrations (0.5 and 50 μM) were projected onto the same PC space to assess whether they could be reliably assigned to binary concentration groups: low (≤5 μM) or high (>5 μM). We found that biofluid test samples generally cluster around each concentration group, however there is some overlap in doxorubicin and epirubicin (Figure 6). To quantify the accuracy of this clustering in the PCA plot, an SVM was used to find the decision boundaries. Prediction models of daunorubicin and idarubicin achieved 100% accuracy with distinct clustering patterns, whereas doxorubicin and epirubicin demonstrated 50% accuracy with substantial overlap in PC1 and PC2 domains, indicating that their concentration-dependent response patterns are not aligned with linear variance (Supplementary Table 3).

**Figure 6:**
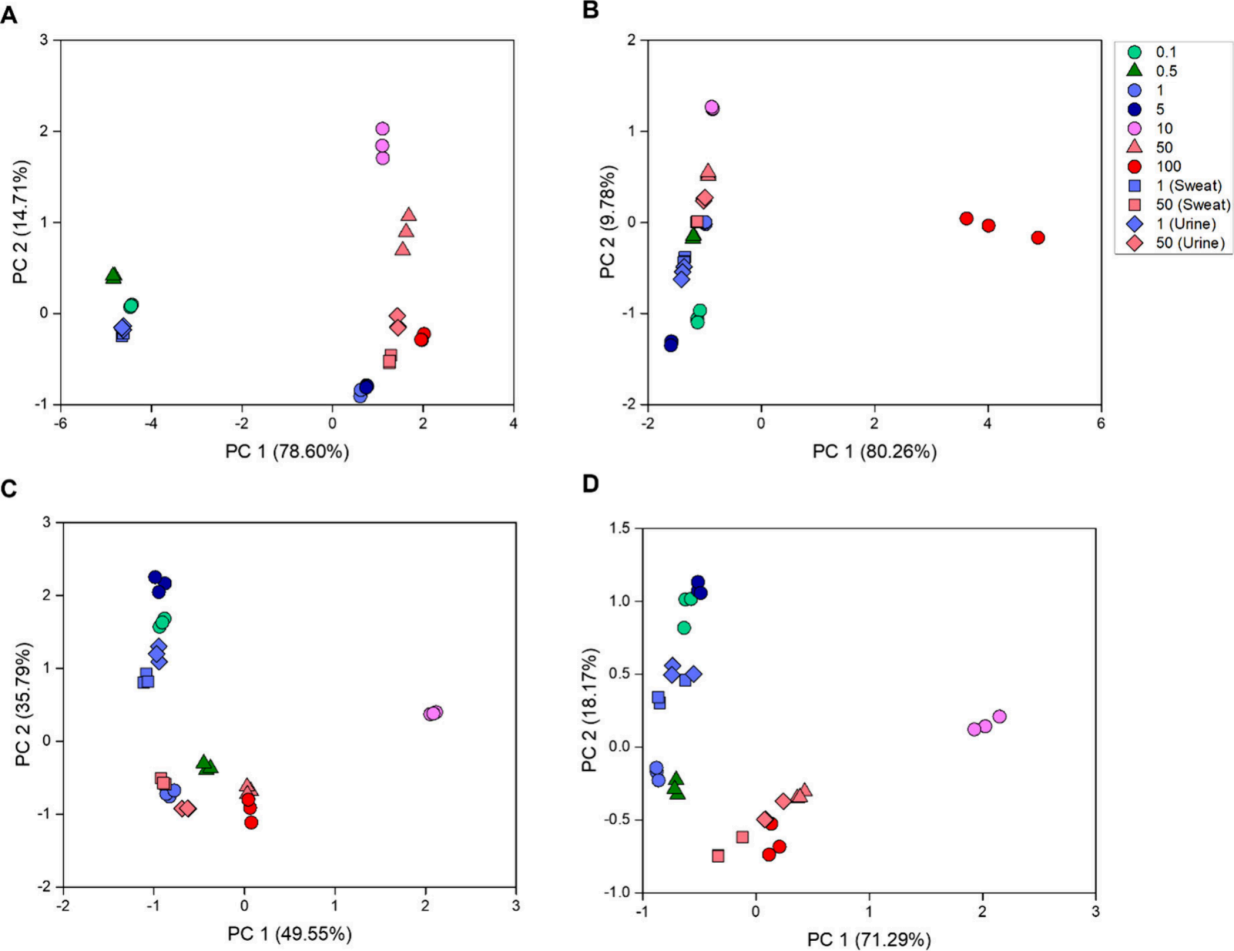
PC score plots for concentration-level discrimination across buffer and biological matrixes: (A) daunorubicin; (B) doxorubicin; (C) epirubicin; (D) idarubicin. PC1 and PC2 domains score plots illustrating the separation of low (≤5 μM) versus high (>5 μM) concentration groups for each anthracycline based on the 48-nanosensor array responses. The PC space was defined using buffer samples at five concentrations as a training set (0.1, 1, 5, 10, and 100 μM; circles). Two intermediate buffer test concentrations (0.5 and 50 μM; triangles) and samples in synthetic sweat (squares) and synthetic urine (diamonds) were projected onto the same PC space. Color denotes concentration: green and blue represent low concentration (≤5 μM), and pink and red represent high concentration (>5 μM) within each anthracycline class.

To identify the nanosensor features most responsible for concentration-dependent variance, principal component loadings were analyzed for the first 10 principal components (Supplementary Table 4). PC loadings quantify the contribution of each feature to the directions of maximum variance, revealing which ssDNA-(*n*,*m*) combinations are most sensitive to concentration changes within each anthracycline class. For daunorubicin, (TAT)_6_-SWCNT exhibited the highest PC1 loadings in their wavelength response, indicating that wavelength changes from these sensors capture the primary axis of concentration-dependent variance. Doxorubicin classification was dominated by (T)_12_ homopolymer sequences, suggesting that polythymine wrapping conformations are particularly sensitive to doxorubicin concentration changes. Epirubicin showed distributed feature importance across multiple sequences, as TTATATTATATT, (GTT)_3_G, and (TAT)_6_, contributed substantially to PC1 variance, reflecting more heterogeneous sensor response patterns. Idarubicin demonstrated strong sequence dependence on GT sequences of varying lengths, indicating that GT-rich motifs provide robust recognition. These loadings reveal distinct sequence-dependent recognition patterns for each anthracycline. Strong sensor sequences revealed by machine learning differ from those discovered in a previous array screen for doxorubicin. In that work, AGAATTACTT, flanked by C-rich ends, was used,^29^ whereas the machine-learning-selected sequences here are either AT- or GT-dominant. Comparing the type of responses observed upon anthracycline interaction, most SWCNTs demonstrated intensity quenching and red shifting across all (*n*,*m*), consistent with what has previously been reported for (GT)_15_-based sensors.^28,30^ The preferential sensors identified through machine learning here demonstrated lower dissociation constants for the top selected daunorubicin (*K*_d_ = 2.2 μM) and doxorubicin (*K*_d_= 12.2 μM) sensors compared to the CB-13 sensor (*K*_d dauno_= 100 μM and *K*_d dox_ = 14 μM).^29^

## Sensor Array Performance Validation in Synthetic Sweat and Urine

We further evaluated our nanosensor array in synthetic sweat and urine as these anthracyclines are regularly found in human waste products (Supplementary Figure 9).^9,63,64^ In this work, we assessed sensor response to 100 nM to 100 μM anthracyclines, with test concentrations in urine and sweat of 500 nm and 50 μM. In patients, anthracyclines are excreted via both the urine and sweat.^63,65,66^ Individual clinical reports have found concentrations of 300 nM idarubicin and 920 nM doxorubicin in the urine of pediatric patients, with another finding as high as 17 μM epirubicin in patient urine^67^ Another report found 1 μM doxorubicin in patient serum.^68^ Additional work has demonstrated accumulation in muscle and other tissue, with an average of 3.37 μM idarubicin across a patient cohort, with a maximum of 19.7 μM.^69^ Other tissues can accumulate concentrations of anthracyclines across a wide range, with concentrations of 200 nm to 52 μM epirubicin found in a cohort of patients, and up to 301 μM found in the tissue of a patient receiving doxuribicin.^70^ Thus, our sensor testing covers most of the upper range, minus outliers, of clinical findings, especially accumulated in tissue. The variability of anthracycline waste and tissue distribution among patients clearly highlights the importance of developing a sensor with a dynamic range of response into high micromolar concentrations.

The same four anthracyclines were spiked into these biological matrixes at concentrations of 1 and 50 μM for all SWCNTs. To evaluate classification performance, the buffer-trained PCA and SVM models were applied to predict concentration levels in biological matrixes (Figure 6). Daunorubicin and idarubicin achieved perfect binary classification accuracy (100%) in distinguishing low (≤5 μM) versus high (>5 μM) concentration groups across both conditions, demonstrating robust discriminatory capability despite the complex matrix composition. In contrast, doxorubicin and epirubicin exhibited substantially lower accuracy (50%), systematically misclassifying high-concentration samples as low concentration while correctly identifying low-concentration samples (Supplementary Table 3). This compound-specific performance discrepancy suggests that doxorubicin and epirubicin generate concentration-dependent response patterns that are either too subtle or nonmonotonic to establish reliable decision boundaries using the current feature set and classification algorithm. The differential performance may arise from structural differences in the anthracycline structure—specifically, the C-14 hydroxyl group present in doxorubicin and epirubicin but absent in daunorubicin and idarubicin—which could alter binding modes, electronic coupling to the SWCNT surface, or susceptibility to matrix interference effects.^71^ Nevertheless, the robust performance for daunorubicin and idarubicin across complex matrixes demonstrates that the nanosensor array maintains clinically relevant discrimination capability for specific anthracyclines in complex biofluids. Further work is required to determine the true LOD of the selected sensors.

## Comparative Analysis to Identify Distinct Optimal Sensors for Each Anthracycline

A comparison of the methods employed for sensor selection revealed no overlap in the preferential sensors [both ssDNA and (*n*,*m*) species] for each anthracycline (Table 1). On the surface, this may be unexpected; however, it is good evidence that each model evaluated separate criteria, such as binding affinity, concentration-dependent sensitivity, and other discriminable factors. Interestingly, we observed that larger-diameter (*n*,*m*) species were consistently selected. Similarly, an analysis of anthracycline adsorption on SWCNT by molecular dynamics found greater adsorption for larger-diameter SWCNT,^72^ which intuitively makes sense as a 1-nm-diameter SWCNT has 25% greater surface area than a 0.8-nm-diameter SWCNT. Above, we noted that smaller-diameter SWCNT species contributed more significant features, although we found that this does not translate to optimal detection. While sensor selection based on the dissociation constant has been used before to determine the selectivity and sensitivity of SWCNT sensors,^15,73^ we found that single-construct data were not consistently well-fit, nor were the dissociation constants as low as in other studies. Because of the diverse pool of ssDNA sequences used here, we postulate that a more systematic length or sequence-based screening pool may improve coherence of predictions. However, increased diversity of sequence space may be valuable for improving sensor function and discriminating between similar molecular structures.

**Table 1:**
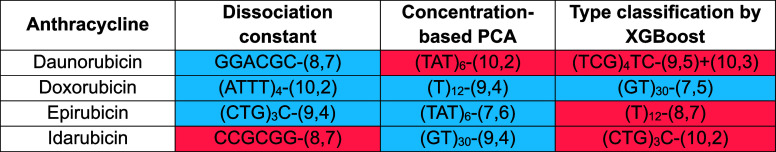
Comparison of ssDNA-(*n*,*m*) Sensor Combinations Identified to Sense Anthracyclines^a^

a Red cells indicate a wavelength shift as the primary means of detection for the SWCNT, while blue cells indicate a change in intensity.

## Conclusions

In this work, we designed a SWCNT nanosensor array and demonstrated the potential clinical applications of several optical nanosensors to detect four anthracycline chemotherapeutics. We implemented the use of high-throughput NIR spectroscopy to analyze fluorescence modulations in the presence of a range of concentrations of each anthracycline. We identified specific ssDNA-(*n*,*m*) constructs that sensitively responded to each anthracycline based on limits of detection and standard nonlinear fits of each binding curve. To enhance anthracycline class detection, supervised machine-learning models were trained and tested, with XGBoost achieving perfect accuracy (100%) in discriminating all four structurally similar anthracyclines. While anthracyclines may not be coadministered in an individual patient, structurally similar metabolites would be present concurrently as each anthracycline, as well as other small molecule therapeutics administered to a patient. PCA-based and SVM-confirmed concentration discrimination successfully separated daunorubicin and idarubicin but showed poor performance for doxorubicin and epirubicin. Validation in synthetic sweat and urine confirmed robust performance, with daunorubicin and idarubicin maintaining 100% concentration classification accuracy despite matrix effects. It is possible that this sensor development methodology could serve as a framework for small molecule detection for which few or no biorecognition elements exist. Assessment of sensor responses in plasma and whole blood will further demonstrate the clinical relevance of our sensors, allowing physicians to reevaluate patient treatment plans to avoid drug-induced toxicity. Overall, we provide a methodology for small molecule sensor development, which furthers the field of therapeutic drug monitoring by developing a diagnostic tool for the anthracycline drug class and demonstrating the potential of SWCNT-based spectral fingerprinting for such applications.

### Brief Methods

Detailed methods are provided in the Supplemental Methods, which describe the synthesis of an optical nanosensor array by separately hybridizing 12 ssDNA sequences with bulk chirality SWCNT, allowing for analysis of seven SWCNT species. Doxorubicin, epirubicin, idarubicin, and daunorubicin were added up to 100 μM and NIR fluorescent spectra obtained. Changes in the intensity and wavelength were obtained, and each construct was analyzed for anthracycline binding affinity prior to PCA. DT, SVM, and XGBoost machine-learning models were used to develop multiclass classification models. SHAP analysis was then applied to quantify the contribution of each spectral feature to the classification output.^74^ Binary classification models were developed and validated in synthetic urine and sweat.

## Supplementary Material



## Data Availability

Primary analyzed data supporting the conclusions of this study are available within the paper and in the Supporting Information. Raw data sets and custom Python code for machine learning are publicly available at Zenodo (DOI: doi.org/10.5281/zenodo.20797573).

## Notes

Representative absorbance and fluorescence spectra of ssDNA-SWCNT constructs in buffer (Supplementary Figure 1), concentration response curves of the (*n*,*m*) species analyzed: (7,6) (SF2); (9,5) + (10,3) (SF3); (10,2) (SF4); (9,4) (SF5); (8,6) (SF6); (8,7) (SF7) (Supplementary Figures 2–7), PCA analysis of anthracyclines at varying concentrations (Supplementary Figure 8), representative fluorescence spectra of ssDNA-SWCNT constructs in 10% synthetic sweat and 10% synthetic urine (Supplementary Figure 9), experimental and theoretical LOD of all sensor constructs (Supplementary Table 1), logistic model dissociation constant values (Supplementary Table 2), performance of SVM models for binary classification (Supplementary Table 3), Top 10 PCA PC1 loadings (Supplementary Table 4) (PDF)
