# Supplementary material for: Optical Spectral Fingerprinting Enables Sensitive Detection of Anthracycline Chemotherapeutics in Synthetic Clinical Biofluids
Source: Nano Lett. 2026 Jul 6;26(28):9122–34. doi: 10.1021/acs.nanolett.6c01777 (PMC13397890; doi:10.1021/acs.nanolett.6c01777)
Supplement: Supplementary file 1 [file nl6c01777_si_001.pdf]

Supplementary Information for:

Optical Spectral Fingerprinting Enables Sensitive Detection of Anthracycline Chemotherapeutics in Synthetic Clinical Biofluids

Atara R. Israel[1 #], Yunjung Kim[2,3 #], Adnan Arnaout[1], Myesha Thahsin[1], Yumna Ahmed[1], Zachary Cohen[1], Amelia Ryan[1], Syeda Rahman[1], Mijin Kim*[2], Ryan M. Williams*[1,4]

[1]Department of Biomedical Engineering, The City College of New York, New York, NY, USA 10031
[2]School of Chemistry and Biochemistry, Georgia Institute of Technology, Atlanta, GA, USA 30332
[3]Department of Chemistry, Hanyang University, Seoul, Republic of Korea 04763
[4]Department of Medicine, Division of Nephrology & Hypertension, Stony Brook University, Stony Brook, NY, USA 11794

[#]These authors equally contributed to the work.
*Co-corresponding authors:
Mkim445@gatech.edu
Ryan.Williams@stonybrookmedicine.edu

Supplementary Methods

Synthesis and characterization of the nanosensor array: As previously described[1], twelve ssDNA sequences (Integrated DNA Technologies; Coralville, IA) were combined, separately, in a 2:1 mass ratio with HiPCo SWCNT (NanoIntegris Technologies; Boisbriand, QC) in a final volume of 0.5 mL with 1X PBS (phosphate-buffered saline). The solution was then probe tip sonicated using a 3 mm stepped probe (Sonics Materials; Newton, CT) at 40% amplitude for one hour on ice. The mixture was then ultracentrifuged using an Optima Max-XP (Beckman Coulter; Brea, CA) at 58,000 X g for one hour. The top 75% of the supernatant was collected and stored at 4°C for future use. Prior to use, excess DNA was removed using a 100 kDa MWCO centrifugal filter (Sigma-Aldrich; St. Louis, MO) at 14,000 g for 15 minutes twice, with a washing step using 200 µL of 1X PBS between runs. The contents of the filter were resuspended in 1X PBS and characterized using absorbance spectroscopy over the range of 400-800 nm. The molar extinction coefficient at 630 nm of 0.036 $L \cdot mg^{-1} \cdot cm^{-1}$ was used to determine the SWCNT concentration of each suspension[2, 3].

Anthracycline addition to nanosensor array: The anthracyclines daunorubicin HCl (LC Laboratories; Woburn, MA), doxorubicin HCl (LC Laboratories), epirubicin HCl (LC Laboratories) and idarubicin HCl (Selleck Chemicals; Houston, TX) were solubilized in a 0.1% dimethyl sulfoxide (DMSO) solution. Additional heating was required to solubilize doxorubicin and epirubicin using a heating block at 60°C for eight minutes with 1000 RPM shaking. Each anthracycline was then added, separately, to 1 mg/L of each of the ssDNA-SWCNT suspensions in triplicate at concentrations of 100, 50, 10, 5, 1, 0.5, and 0.1 µM. These were incubated in a Corning UV-transparent half-area 96-well plate (Fisher Scientific; Hampton, NH) at a final volume of 150 µL in 0.1% DMSO for one hour at room temperature.

High throughput NIR fluorescence spectroscopy: Fluorescence spectroscopy of the nanosensor array before and after anthracycline addition was obtained using a near-infrared plate reader (Photon, Etc.; Montreal, QC), with emission spectra acquired from 900-1600 nm after sequential laser excitation of 655 nm and 730 nm of 1700 mW and exposure time of 500 ms. Each laser excitation source was used to obtain spectra from (*n,m*) species: [(7,5), (7,6), and (9,5)+(10,3)], and [(10,2), (9,4), (8,6), and (8,7)], respectively.

Data analysis: Changes in intensity and wavelength of SWCNT species were analyzed using a custom MATLAB code as described in our prior work[4]. Each full spectra (900-1400 nm) was imported into MATLAB and a blank baseline spectra was subtracted, after which user-defined peaks (manual selection of lower and upper wavelength boundaries) were selected in bulk for each experiment. We selected the 3 most prominent peaks following 655 nm excitation and the four most prominent peak following 730 nm excitation. Each user-defined peak was then fit to a pseudo-Voigt profile to reduce noise. The MATLAB code reported the center wavelength of each fit spectra as well as the baseline-subtracted maximum intensity (non-normalized) and the goodness of fit (R^2). Only R^2 values of peak fits greater than 0.9 were used in further data analysis on the basis that poor fits indicate a lack of defined fluorescence spectra. Changes in center wavelength and maximum intensity were reported relative to the control (no anthracycline). The dissociation constants (K_d) were derived from the spectral responses of SWCNTs using a standard non-cooperative binding model: $response = baseline + \frac{\Delta \max * [analyte]}{K_d + [analyte]}$ where response is the signal obtained (either change in center wavelength or intensity) at a given analyte concentration, baseline is the response of the control, Δmax is the maximum response observed, [analyte] is the analyte concentration, and K_d is the analyte concentration which elicits 50% of the maximum response. The fittings were performed in OriginPro. Statistical significance in fluorescence change was determined by a one-way ANOVA with Dunnett's post hoc analysis using OriginPro. Principal component analysis (PCA) and machine learning models were implemented for classification in Python 3.12.2 using the *scikit-learn* 1.7.1 package[5, 6].

Multi-class classification of anthracycline type: Three supervised machine learning models, Decision Tree (DT), support vector machine (SVM), and eXtreme Gradient Boosting (XGBoost, XGB), implemented in the *scikit-learn* library, were used to develop multi-class classification models. The training set comprised spectral response data acquired at four concentration levels (0.1, 1, 10, and 100 μM). Model generalization and predictive accuracy were assessed using an independent test set comprising intermediate concentrations (0.5, 5, and 50 μM). Hyperparameter optimization was conducted using Bayesian optimization with *k*-fold cross-validation (*k* = 3) on the training set to identify the optimal model configuration. SHAP (SHapley Additive exPlanations) analysis was then applied to quantify the contribution of each spectral feature to the classification output[7].

Development and validation of concentration-based binary classification models: The training dataset was constructed exclusively from 1X PBS containing 0.1% DMSO at five anthracycline concentrations (0.1, 1, 5, 10, 100 μM). Test datasets were used as independent samples with intermediate concentrations (0.5, 50 μM). Concentrations ≤ 5 μM were labeled as "Low" and those > 5 μM as "High". The model development and optimization process were the same as described

in the multi-class classification models. Performance of the optimized models were assessed using 10% solutions of synthetic urine (Sigma-Aldrich; St. Louis, MO) and synthetic sweat (Sigma-Aldrich, St. Louis, MO) containing 1 mg/L of each sensor construct. The solutions were spiked with each anthracycline at concentrations of 1 and 50 µM. Sensor responses from each spiked medium were acquired and processed as described above. PCA was fitted on the training data only, and the resulting components were applied to both the training and test sets to avoid data leakage. These PCA-transformed features within the PC domain were used to discriminate between low- and high-concentration classes. Furthermore, SVM classification was performed on the PC domain to evaluate PCA-based discrimination accuracy. This validation quantified model robustness against matrix effects and compositional variability inherent in biofluids.

Supplementary Figures

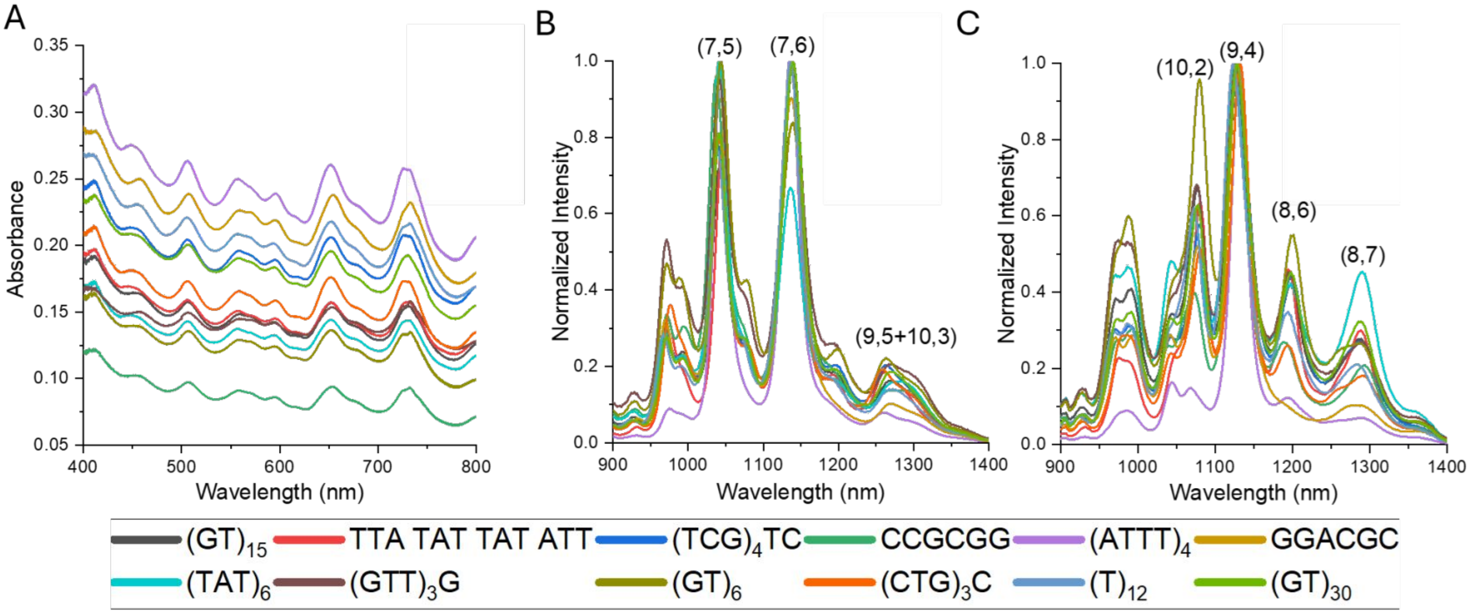

Supplementary Figure 1. **Characterization of SWCNT sensor constructs.** A) Representative UV-Vis absorbance spectra. B) Representative fluorescence spectra of SWCNT constructs at excitation wavelength 655 nm. C) Representative fluorescence spectra of SWCNT constructs at excitation wavelength 730 nm.

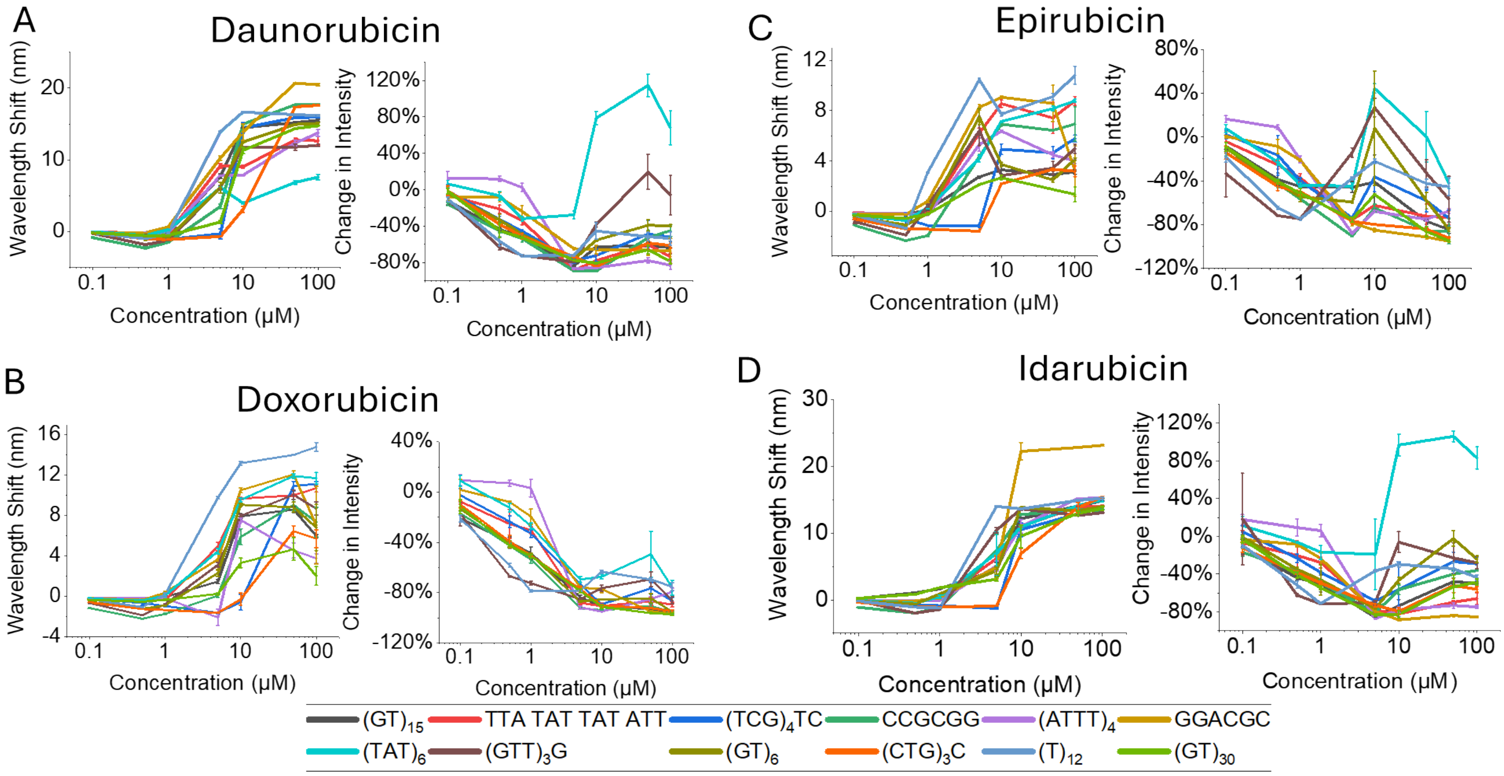

Supplementary Figure 2. Concentration response curves of A) daunorubicin, B) doxorubicin, C) epirubicin, and D) idarubicin of all sensor constructs for the (7,6) chirality.

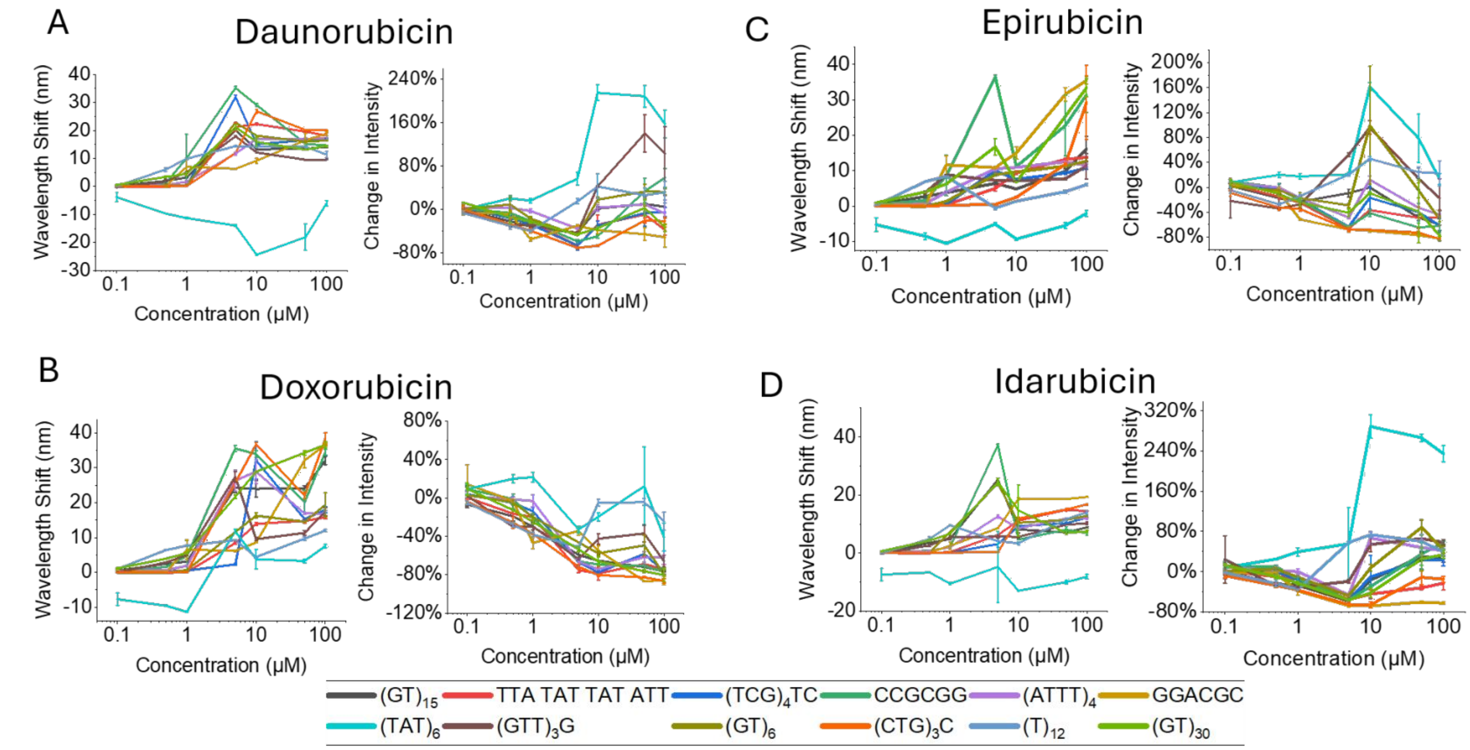

Supplementary Figure 3. Concentration response curves of A) daunorubicin, B) doxorubicin, C) epirubicin, and D) idarubicin of all sensor constructs for the (9,5+10,3) chiralities.

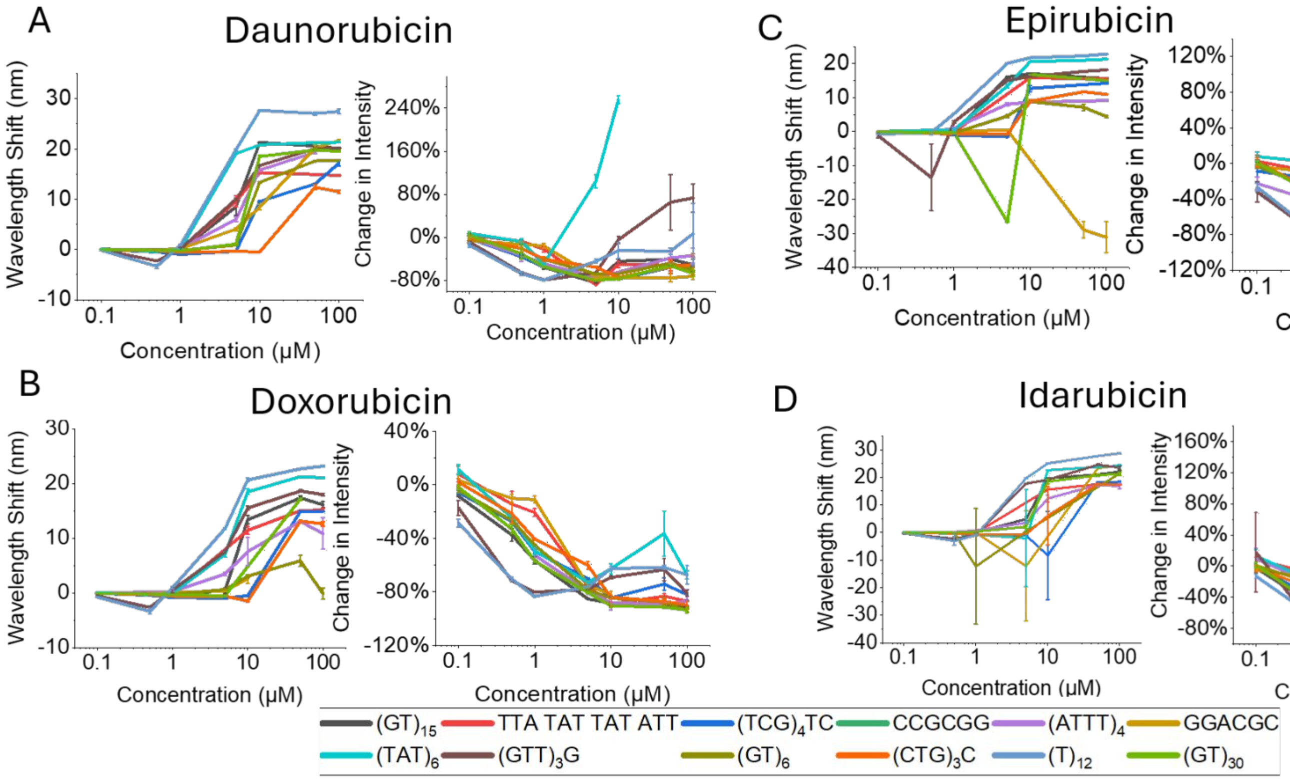

Supplementary Figure 4. Concentration response curves of A) daunorubicin, B) doxorubicin, C) epirubicin, and D) idarubicin of all sensor constructs for the (10,2) chirality.

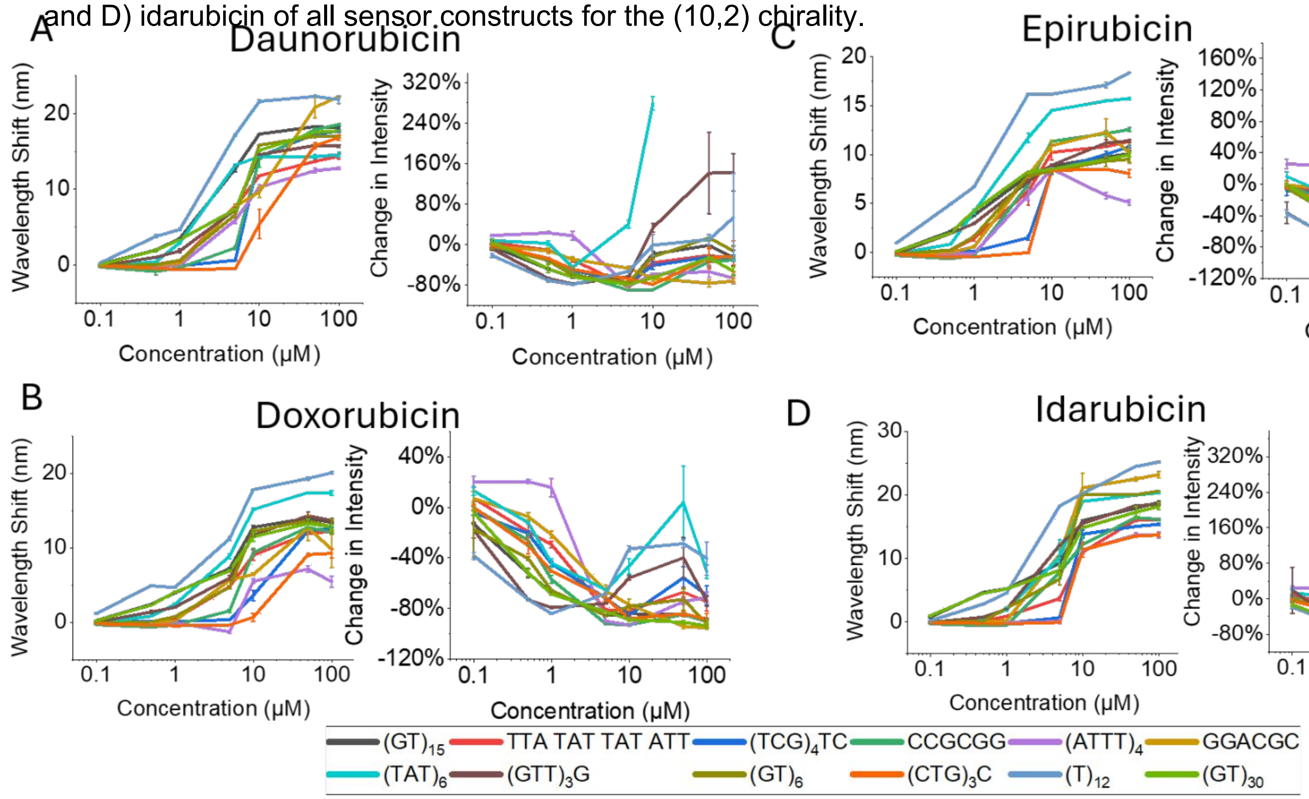

Supplementary Figure 5. Concentration response curves of A) daunorubicin, B) doxorubicin, C) epirubicin, and D) idarubicin of all sensor constructs for the (9,4) chirality.

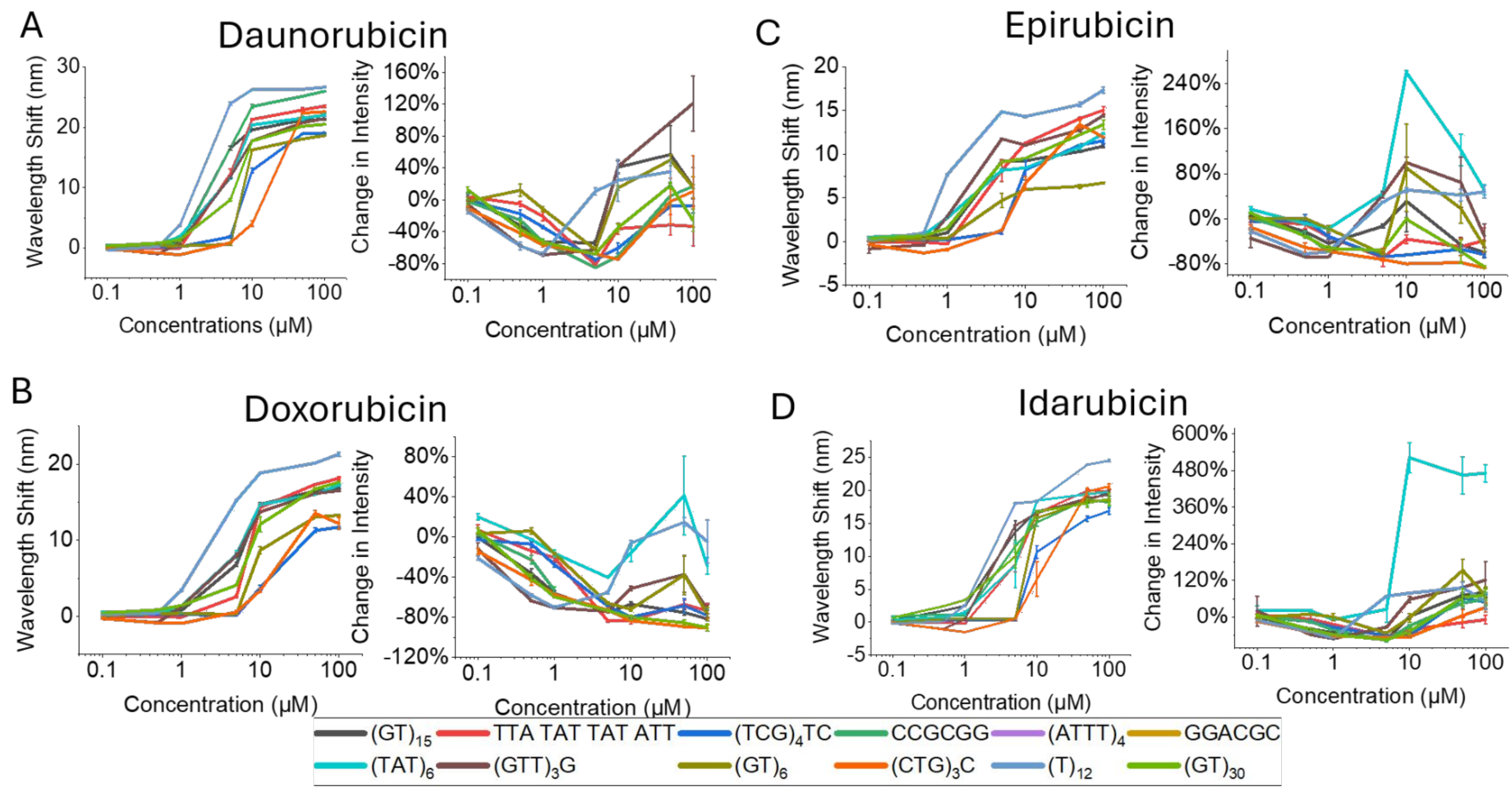

Supplementary Figure 6. Concentration response curves of A) daunorubicin, B) doxorubicin, C) epirubicin, and D) idarubicin of all sensor constructs for the (8,6) chirality.

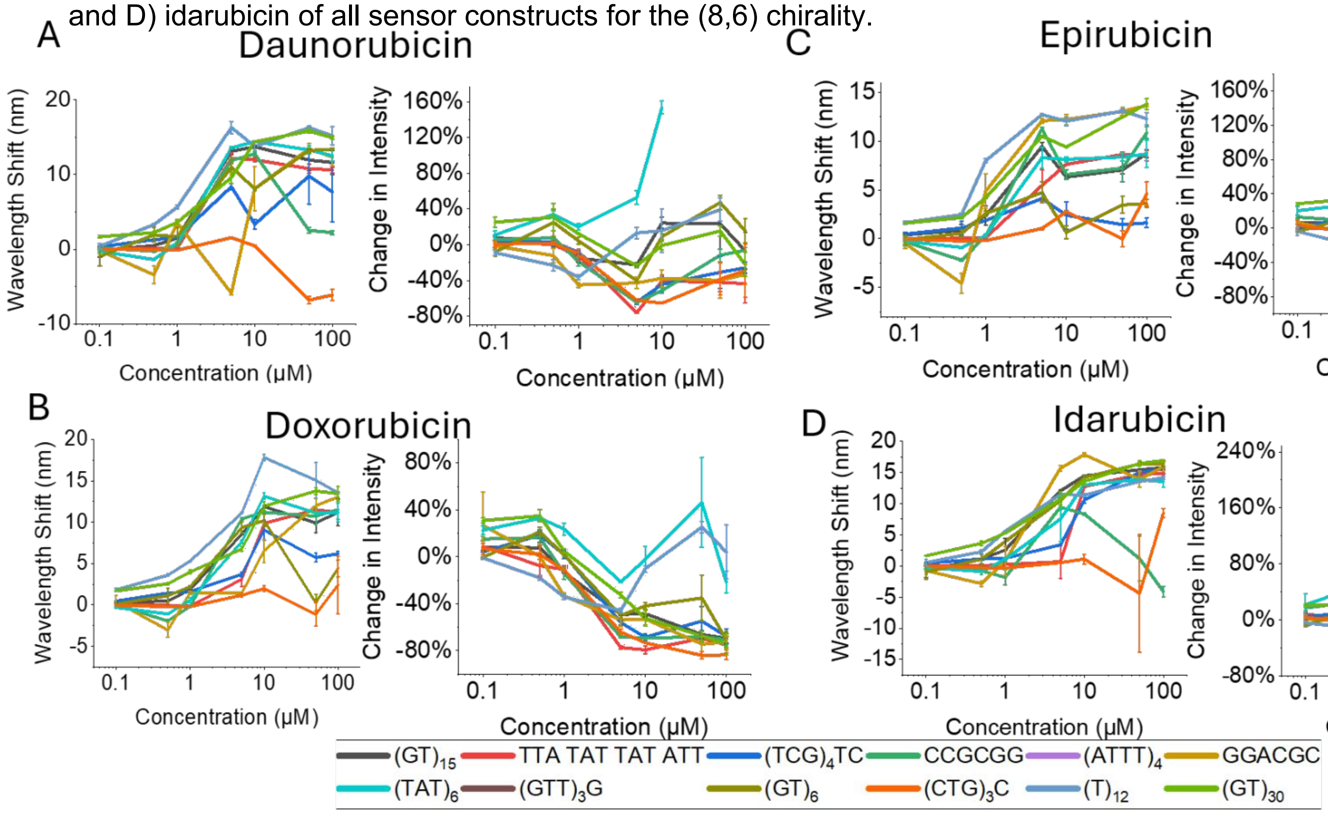

Supplementary Figure 7. Concentration response curves of A) daunorubicin, B) doxorubicin, C) epirubicin, and D) idarubicin of all sensor constructs for the (8,7) chirality.

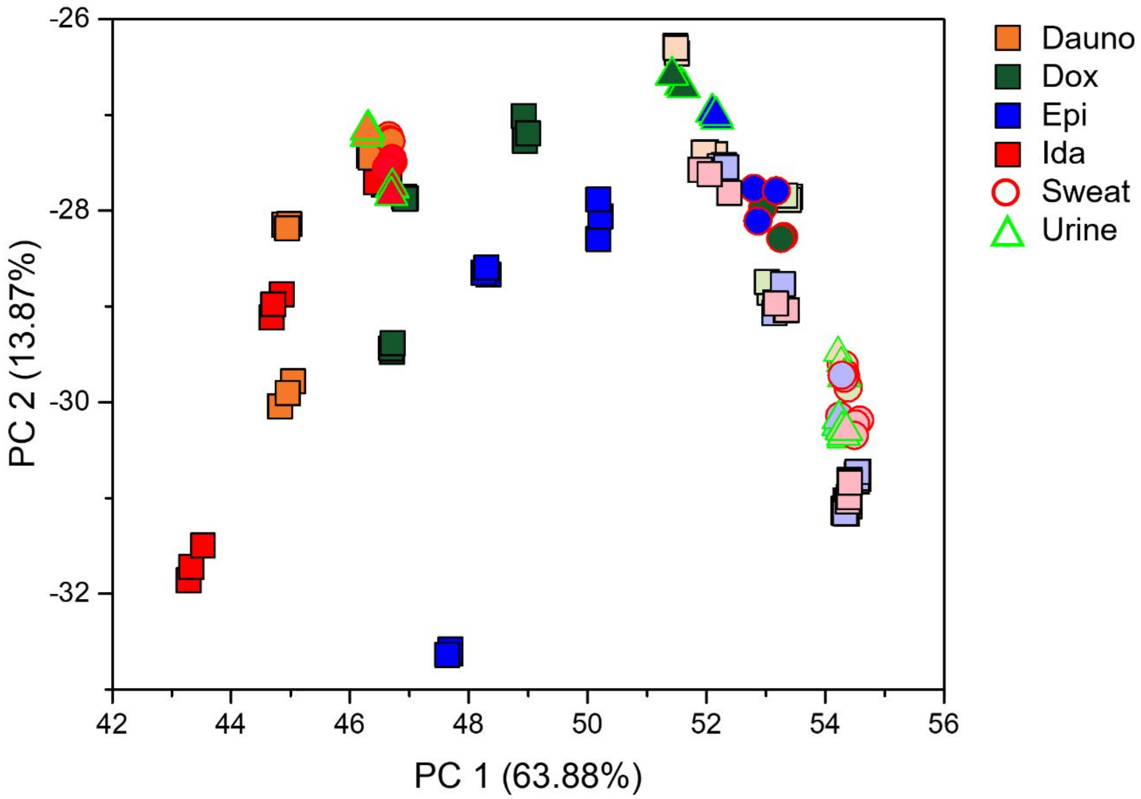

Supplementary Figure 8. PCA plot of anthracycline classes at different concentrations. Color intensity denotes concentration (bright ≤ 5 μM; dark > 5 μM). Colors indicate anthracycline type: daunorubicin (orange), doxorubicin (green), epirubicin (blue), and idarubicin (red). Shapes denote matrices: buffer (square), synthetic sweat (circle), and synthetic urine (triangle).

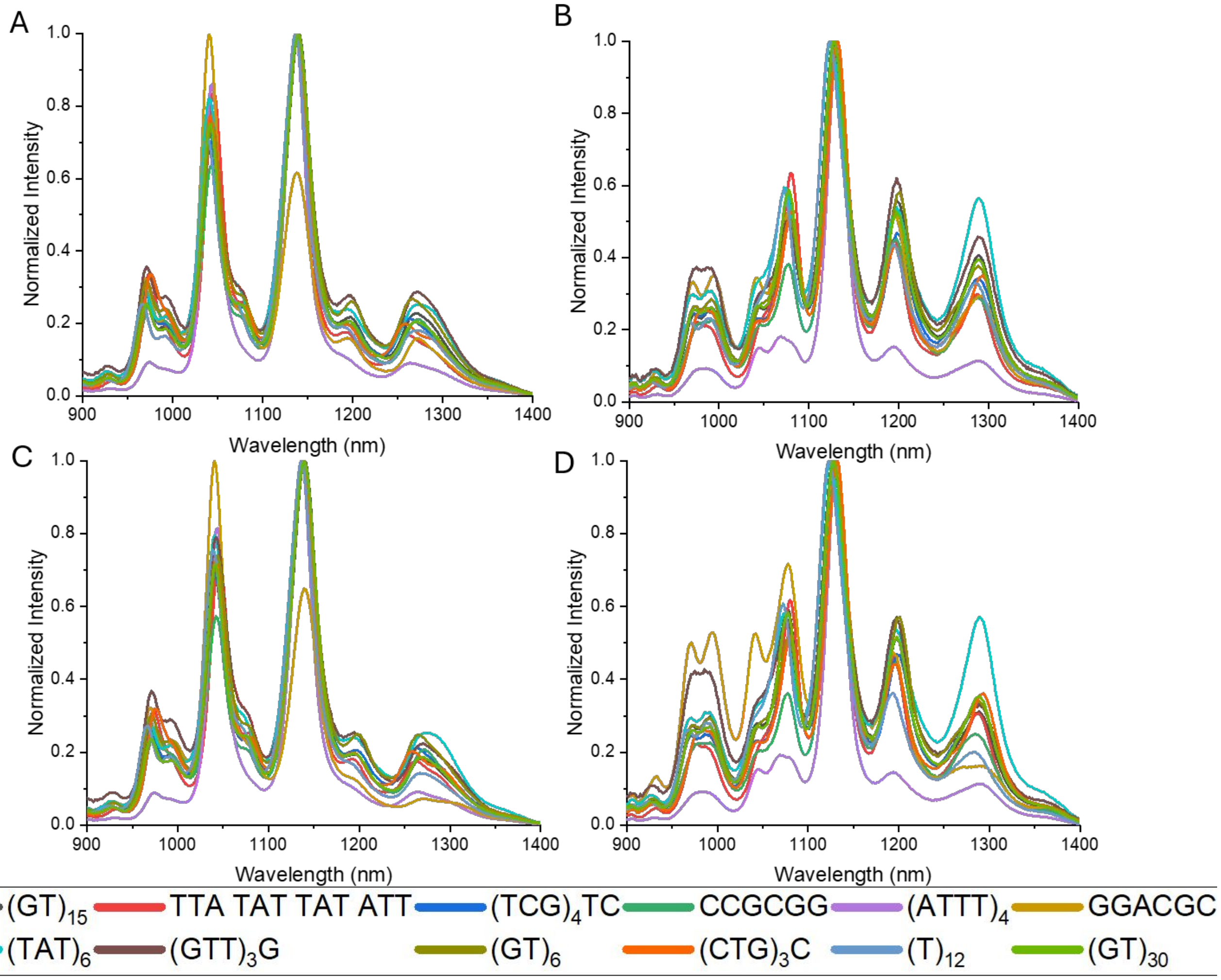

Supplementary Figure 9. Representative fluorescence spectra of all SWCNT constructs in A) 10% synthetic sweat at an excitation of 655 nm, B) 10% synthetic sweat at an excitation of 730 nm, C) 10% synthetic urine at an excitation of 655 nm, and D) 10% urine at an excitation of 730 nm.

Supplementary Tables

Supplementary Table 1. Theoretical limits of detection (LOD) (calculated as $3^*\sigma_{control}$) for all sensor constructs compared to the experimentally determined LOD (based on ANOVA $p<0.05$).

Sequence	Chirality	Theoretical LOD_wv	Experimental LOD_wv	Theoretical LOD_int	Experimental LOD_int
$(GT)_{15}$	(10,2)	0.12861	Dauno - 5<br>Dox – 10<br>Epi - 5<br>Ida - 5	0.596	Dauno – 0.1<br>Dox – 0.1<br>Epi – 0.5<br>Ida - 0.1
$(GT)_{15}$	(7,5)	0.3289	Dauno - 5<br>Dox - 5<br>Epi - 5	1.976	Dauno – 0.1<br>Dox – 0.1<br>Epi – 50

			Ida – 5		Ida – 0.1
(GT)$_{15}$	(7,6)	1.39748	Dauno - 5<br>Dox - 5<br>Epi - 5<br>Ida – 0.5	1.634	Dauno – 0.5<br>Dox – 0.1<br>Epi – 0.5<br>Ida – 0.5
(GT)$_{15}$	(8,6)	0.19164	Dauno – 1<br>Dox - 5<br>Epi - 1<br>Ida - 1	0.703	Dauno – n/a<br>Dox – 0.1<br>Epi - 50<br>Ida - 100
(GT)$_{15}$	(8,7)	3.31338	Dauno – 5<br>Dox - 5<br>Epi - 5<br>Ida - 5	1.151	Dauno - 100<br>Dox – 0.1<br>Epi - 50<br>Ida – n/a
(GT)$_{15}$	(9,4)	0.44131	Dauno – 0.5<br>Dox – 0.5<br>Epi – 0.5<br>Ida – 0.5	0.677	Dauno – 0.5<br>Dox – 0.1<br>Epi - 50<br>Ida – n/a
(GT)$_{15}$	(9,5+10,3)	0.6772	Dauno – 0.5<br>Dox - 5<br>Epi - 5<br>Ida – 0.5	3.129	Dauno – 0.1<br>Dox – 0.1<br>Epi – 0.1<br>Ida – 0.1
TTA TAT TAT ATT	(10,2)	0.06004	Dauno - 5<br>Dox - 5<br>Epi – n/a<br>Ida - 5	1.325	Dauno - 5<br>Dox - 5<br>Epi – 5<br>Ida – 10
TTA TAT TAT ATT	(7,5)	0.36057	Dauno - 5<br>Dox - 5<br>Epi - 5<br>Ida - 5	1.183	Dauno - 5<br>Dox - 5<br>Epi – n/a<br>Ida – n/a
TTA TAT TAT ATT	(7,6)	1.49272	Dauno - 5<br>Dox - 5<br>Epi - 5<br>Ida - 5	1.537	Dauno - 5<br>Dox - 5<br>Epi - 5<br>Ida - 5
TTA TAT TAT ATT	(8,6)	0.26255	Dauno - 5<br>Dox - 5<br>Epi - 5<br>Ida - 5	1.54	Dauno – n/a<br>Dox - 5<br>Epi – n/a<br>Ida – n/a
TTA TAT TAT ATT	(8,7)	0.37601	Dauno - 5<br>Dox - 5<br>Epi - 5<br>Ida - 10	1.514	Dauno - 100<br>Dox - 5<br>Epi – n/a<br>Ida – n/a
TTA TAT TAT ATT	(9,4)	0.31263	Dauno - 5<br>Dox - 5<br>Epi - 5<br>Ida - 1	1.387	Dauno – n/a<br>Dox - 5<br>Epi – n/a<br>Ida – n/a
TTA TAT TAT ATT	(9,5+10,3)	0.24238	Dauno - 5<br>Dox - 5<br>Epi - 5<br>Ida - 5	1.849	Dauno – n/a<br>Dox -5<br>Epi – n/a<br>Ida – n/a
(TCG)$_4$TC	(10,2)	0.31444	Dauno – 0.5	4.235	Dauno - n/a

			Dox – 0.5<br>Epi - 1<br>Ida - 1		Dox - n/a<br>Epi - n/a<br>Ida - n/a
$(TCG)_4TC$	(7,5)	0.78879	Dauno - 10<br>Dox - 50<br>Epi - 5<br>Ida - 10	4.814	Dauno – n/a<br>Dox - n/a<br>Epi - n/a<br>Ida - n/a
$(TCG)_4TC$	(7,6)	0.54083	Dauno - 10<br>Dox - 50<br>Epi - 10<br>Ida - 10	4.878	Dauno - n/a<br>Dox - n/a<br>Epi - n/a<br>Ida - n/a
$(TCG)_4TC$	(8,6)	0.10638	Dauno - 5<br>Dox - 10<br>Epi - 10<br>Ida - 10	0.111	Dauno - n/a<br>Dox - 5<br>Epi - 5<br>Ida - 50
$(TCG)_4TC$	(8,7)	0.53573	Dauno - 50<br>Dox - 1<br>Epi - n/a<br>Ida – 0.5	0.103	Dauno - n/a<br>Dox – 0.1<br>Epi – 0.1<br>Ida - n/a
$(TCG)_4TC$	(9,4)	0.16406	Dauno - 5<br>Dox - 10<br>Epi - 5<br>Ida - 10	0.108	Dauno - n/a<br>Dox - 1<br>Epi - n/a<br>Ida - 50
$(TCG)_4TC$	(9,5+10,3)	1.63363	Dauno - 5<br>Dox - 10<br>Epi - 5<br>Ida - 5	5.002	Dauno - n/a<br>Dox - n/a<br>Epi - n/a<br>Ida - n/a
CCG CGG	(7,5)	0.2427	Dauno - 10<br>Dox - 10<br>Epi – 0.5<br>Ida - 10	1.366	Dauno - n/a<br>Dox - 5<br>Epi - 50<br>Ida - n/a
CCG CGG	(7,6)	0.38861	Dauno – 0.1<br>Dox - 10<br>Epi – 0.5<br>Ida - 5	1.411	Dauno - n/a<br>Dox - 1<br>Epi - 1<br>Ida - n/a
CCG CGG	(8,6)	0.79	Dauno - 5<br>Dox - 10<br>Epi – 0.1<br>Ida – 0.1	0.2	Dauno - 1<br>Dox - 10<br>Epi - 1<br>Ida - 50
CCG CGG	(8,7)	0.56565	Dauno - 5<br>Dox - 5<br>Epi - 5<br>Ida - 100	0.212	Dauno - 100<br>Dox - 5<br>Epi - 5<br>Ida - 50
CCG CGG	(9,4)	0.08478	Dauno - 5<br>Dox - 5<br>Epi - 5<br>Ida - 5	0.183	Dauno – 0.1<br>Dox – 0.1<br>Epi - 1<br>Ida – 0.1
CCG CGG	(9,5+10,3)	0.9179	Dauno - 5<br>Dox - 5<br>Epi - 50	1.381	Dauno - 100<br>Dox - 5<br>Epi - n/a

			Ida – 0.1		Ida - 100
$(ATTT)_4$	(10,2)	0.31884	Dauno – 0.1<br>Dox - 5<br>Epi – 0.1<br>Ida – 0.1	1.506	Dauno - n/a<br>Dox - n/a<br>Epi - n/a<br>Ida - n/a
$(ATTT)_4$	(7,5)	0.26073	Dauno - 10<br>Dox - 10<br>Epi - 5<br>Ida - 10	0.896	Dauno - 5<br>Dox - 10<br>Epi - 50<br>Ida - 10
$(ATTT)_4$	(9,5+10,3)	0.21447	Dauno - 1<br>Dox - 5<br>Epi – 0.5<br>Ida – 0.5	1.064	Dauno - n/a<br>Dox - n/a<br>Epi - n/a<br>Ida - 10
GGA CGC	(10,2)	0.17449	Dauno - 5<br>Dox - 10<br>Epi - 50<br>Ida - 5	1.472	Dauno - 5<br>Dox - 5<br>Epi - 50<br>Ida - 5
GGA CGC	(7,5)	0.41245	Dauno - 5<br>Dox - 10<br>Epi - 5<br>Ida - 10	2.066	Dauno - n/a<br>Dox - 50<br>Epi - 50<br>Ida - n/a
GGA CGC	(7,6)	1.21187	Dauno – 5<br>Dox – 10<br>Epi - 5<br>Ida - 5	2.128	Dauno - n/a<br>Dox - 50<br>Epi - 50<br>Ida - n/a
GGA CGC	(8,7)	0.27673	Dauno - 10<br>Dox - 1<br>Epi - 1<br>Ida - 1	0.613	Dauno - n/a<br>Dox - 50<br>Epi - 50<br>Ida - 100
GGA CGC	(9,4)	0.21677	Dauno - 5<br>Dox - 5<br>Epi - 5<br>Ida - 5	1.51	Dauno - 10<br>Dox - 5<br>Epi - 5<br>Ida - 5
GGA CGC	(9,5+10,3)	1.24989	Dauno - 1<br>Dox - 1<br>Epi - 1<br>Ida - 1	2.587	Dauno - n/a<br>Dox - n/a<br>Epi - n/a<br>Ida - n/a
$(TAT)_6$	(10,2)	0.08146	Dauno - 5<br>Dox - 5<br>Epi - 5<br>Ida – 0.5	0.5	Dauno - 10<br>Dox - 1<br>Epi - n/a<br>Ida - 10
$(TAT)_6$	(7,5)	0.01726	Dauno - 1<br>Dox - 1<br>Epi – 0.5<br>Ida - 5	0.716	Dauno - 10<br>Dox – 0.5<br>Epi - 10<br>Ida - 10
$(TAT)_6$	(7,6)	0.14425	Dauno - 5<br>Dox - 5<br>Epi – 0.5<br>Ida - 5	0.728	Dauno - 10<br>Dox - 5<br>Epi - 1<br>Ida - 10
$(TAT)_6$	(8,6)	0.31734	Dauno - 1	0.536	Dauno - 10

			Dox - 1<br>Epi - 1<br>Ida - 5		Dox - 100<br>Epi - 5<br>Ida - 10
$(TAT)_6$	(8,7)	1.69853	Dauno - 1<br>Dox - 1<br>Epi - 5<br>Ida - 5	0.57	Dauno - 10<br>Dox - n/a<br>Epi - 5<br>Ida - 10
$(TAT)_6$	(9,4)	0.27307	Dauno - 1<br>Dox – 0.5<br>Epi – 0.5<br>Ida - 5	0.565	Dauno - 10<br>Dox - n/a<br>Epi - n/a<br>Ida - 10
$(TAT)_6$	(9,5+10,3)	7.44552	Dauno - n/a<br>Dox - 1<br>Epi - n/a<br>Ida - n/a	0.23	Dauno - 5<br>Dox - n/a<br>Epi - n/a<br>Ida - 10
$(GTT)_3G$	(10,2)	1.48603	Dauno - 5<br>Dox - 5<br>Epi - 5<br>Ida - 5	0.49	Dauno - n/a<br>Dox – 0.1<br>Epi - 100<br>Ida - n/a
$(GTT)_3G$	(7,5)	0.96204	Dauno - 1<br>Dox - 1<br>Epi - 1<br>Ida - 1	0.6	Dauno - 50<br>Dox - 100<br>Epi - n/a<br>Ida - 10
$(GTT)_3G$	(7,6)	0.46275	Dauno - 5<br>Dox - 5<br>Epi - 1<br>Ida - 5	0.503	Dauno - 50<br>Dox - 100<br>Epi - n/a<br>Ida - n/a
$(GTT)_3G$	(8,6)	0.06248	Dauno – 0.5<br>Dox – 0.5<br>Epi - 1<br>Ida - 5	0.403	Dauno - 50<br>Dox – 0.5<br>Epi - n/a<br>Ida - 50
$(GTT)_3G$	(9,4)	0.02869	Dauno – 0.5<br>Dox – 0.5<br>Epi – 0.1<br>Ida - 1	0.479	Dauno - 50<br>Dox – 0.5<br>Epi - n/a<br>Ida - n/a
$(GTT)_3G$	(9,5+10,3)	2.79266	Dauno – 0.5<br>Dox – 0.5<br>Epi – 0.5<br>Ida – 0.5	0.466	Dauno - 10<br>Dox - n/a<br>Epi - n/a<br>Ida - 10
$(GT)_6$	(10,2)	0.44529	Dauno - 1<br>Dox - n/a<br>Epi - 5<br>Ida - 100	0.999	Dauno - 100<br>Dox - 5<br>Epi - 100<br>Ida - n/a
$(GT)_6$	(7,5)	0.97191	Dauno - 5<br>Dox - 5<br>Epi - 1<br>Ida - 5	0.977	Dauno - n/a<br>Dox - 5<br>Epi - n/a<br>Ida - n/a
$(GT)_6$	(7,6)	0.40084	Dauno - 5<br>Dox - 5<br>Epi - 5	0.954	Dauno - n/a<br>Dox - 5<br>Epi - n/a

			Ida - 1		Ida - n/a
$(GT)_6$	(8,6)	0.39931	Dauno - 5<br>Dox - 10<br>Epi - 5<br>Ida - 10	1.065	Dauno - 50<br>Dox - 100<br>Epi - n/a<br>Ida - 50
$(GT)_6$	(8,7)	1.86924	Dauno - 5<br>Dox - 100<br>Epi - 50<br>Ida - 1	0.267	Dauno - 10<br>Dox - 100<br>Epi - n/a<br>Ida - 50
$(GT)_6$	(9,4)	0.62225	Dauno - 1<br>Dox - 5<br>Epi - 1<br>Ida - 1	1.012	Dauno - n/a<br>Dox - 1<br>Epi - n/a<br>Ida - n/a
$(GT)_6$	(9,5+10,3)	1.22519	Dauno - 5<br>Dox - 10<br>Epi - 1<br>Ida - 5	0.886	Dauno - 10<br>Dox - 100<br>Epi - n/a<br>Ida - 10
$(CTG)_3C$	(10,2)	0.08326	Dauno - 50<br>Dox - 1<br>Epi – 0.5<br>Ida - 50	0.25	Dauno - 5<br>Dox - 1<br>Epi - 1<br>Ida - 1
$(CTG)_3C$	(7,5)	0.0253	Dauno - 50<br>Dox - 50<br>Epi - 10<br>Ida - 50	0.11	Dauno - 1<br>Dox - 5<br>Epi - 5<br>Ida - 100
$(CTG)_3C$	(7,6)	0.10945	Dauno – 0.5<br>Dox - 50<br>Epi – 0.1<br>Ida - 10	0.104	Dauno – 0.1<br>Dox – 0.1<br>Epi – 0.5<br>Ida – 0.5
$(CTG)_3C$	(8,6)	0.3439	Dauno – 0.5<br>Dox - 10<br>Epi – 0.5<br>Ida - 10	0.189	Dauno - n/a<br>Dox – 0.5<br>Epi – 0.5<br>Ida - 100
$(CTG)_3C$	(8,7)	1.41223	Dauno - 50<br>Dox - n/a<br>Epi - 100<br>Ida - n/a	0.173	Dauno - n/a<br>Dox - 5<br>Epi - 5<br>Ida - n/a
$(CTG)_3C$	(9,4)	0.13168	Dauno - 10<br>Dox - 50<br>Epi - 10<br>Ida - 10	0.241	Dauno - n/a<br>Dox – 0.1<br>Epi – 0.5<br>Ida - n/a
$(CTG)_3C$	(9,5+10,3)	0.10956	Dauno - 5<br>Dox - 10<br>Epi - 100<br>Ida - 10	0.117	Dauno - n/a<br>Dox – 0.1<br>Epi – 0.5<br>Ida - n/a
$(T)_{12}$	(10,2)	0.4144	Dauno - 5<br>Dox – 0.1<br>Epi - 1<br>Ida - 5	1.447	Dauno - n/a<br>Dox – 0.5<br>Epi - 100<br>Ida - n/a
$(T)_{12}$	(7,5)	0.18774	Dauno – 0.5	0.239	Dauno - n/a

			Dox - 1<br>Epi – 0.5<br>Ida - 1		Dox – 0.5<br>Epi - n/a<br>Ida - n/a
$(T)_{12}$	(7,6)	0.53042	Dauno - 5<br>Dox - 5<br>Epi – 0.5<br>Ida – 0.5	2.643	Dauno - n/a<br>Dox - n/a<br>Epi - n/a<br>Ida - n/a
$(T)_{12}$	(8,6)	0.50043	Dauno - 1<br>Dox - 1<br>Epi – 0.5<br>Ida - 1	1.501	Dauno - n/a<br>Dox - n/a<br>Epi - n/a<br>Ida - n/a
$(T)_{12}$	(8,7)	4.20116	Dauno – 0.5<br>Dox – 0.5<br>Epi – 0.5<br>Ida – 0.5	1.527	Dauno - n/a<br>Dox - n/a<br>Epi - n/a<br>Ida - n/a
$(T)_{12}$	(9,4)	0.16061	Dauno – 0.5<br>Dox – 0.1<br>Epi – 0.1<br>Ida – 0.5	1.509	Dauno - n/a<br>Dox - 100<br>Epi - n/a<br>Ida - n/a
$(T)_{12}$	(9,5+10,3)	1.53304	Dauno – 0.5<br>Dox – 0.1<br>Epi - 10<br>Ida – 0.5	2.771	Dauno - n/a<br>Dox - n/a<br>Epi - n/a<br>Ida - n/a
$(GT)_{30}$	(10,2)	0.16727	Dauno - 5<br>Dox - n/a<br>Epi – 0.5<br>Ida - 5	0.308	Dauno - 1<br>Dox - 1<br>Epi - 50<br>Ida - n/a
$(GT)_{30}$	(7,5)	0.08858	Dauno - 5<br>Dox - 5<br>Epi – 0.5<br>Ida - 10	0.452	Dauno - 5<br>Dox - 1<br>Epi - 10<br>Ida - n/a
$(GT)_{30}$	(7,6)	0.78338	Dauno - 5<br>Dox - 10<br>Epi - 5<br>Ida - 5	0.5	Dauno – 0.1<br>Dox – 0.5<br>Epi - 1<br>Ida - 1
$(GT)_{30}$	(8,6)	0.27241	Dauno – 0.5<br>Dox – 5<br>Epi – 0.5<br>Ida – 0.5	0.31	Dauno - n/a<br>Dox – 0.1<br>Epi - 1<br>Ida - 50
$(GT)_{30}$	(8,7)	3.62661	Dauno – 0.1<br>Dox – 0.1<br>Epi – 0.1<br>Ida – 0.1	0.313	Dauno - n/a<br>Dox - 10<br>Epi - 10<br>Ida - 50
$(GT)_{30}$	(9,4)	0.07942	Dauno – 0.5<br>Dox – 0.5<br>Epi – 0.5<br>Ida – 0.5	0.31	Dauno - 100<br>Dox – 0.1<br>Epi - 50<br>Ida - n/a
$(GT)_{30}$	(9,5+10,3)	0.83466	Dauno – 0.5<br>Dox – 0.5<br>Epi - 1	0.54	Dauno - n/a<br>Dox - 5<br>Epi - 50

			Ida - n/a		Ida - 50

Supplementary Table 2. Logistic model results of wavelength and intensity-induced anthracycline responses

**Sequence**	**Anthracycline**	***(n,m)* species**	**K_d_wv**	**K_d_int**
$(GT)_{15}$	Dauno	(10,2)	4.084	Fit failed
$(GT)_{15}$	Dauno	(7,5)	4.223	Fit failed
$(GT)_{15}$	Dauno	(7,6)	3.965	0.182
$(GT)_{15}$	Dauno	(8,6)	2.566	Fit failed
$(GT)_{15}$	Dauno	(8,7)	1.455	Fit failed
$(GT)_{15}$	Dauno	(9,4)	2.375	Fit failed
$(GT)_{15}$	Dauno	(9,5+10,3)	Fit failed	Fit failed
$(GT)_{15}$	Dox	(10,2)	10.21	0.583
$(GT)_{15}$	Dox	(7,5)	7.394	1.477
$(GT)_{15}$	Dox	(7,6)	4.818	1.206
$(GT)_{15}$	Dox	(8,6)	5.058	0.307
$(GT)_{15}$	Dox	(8,7)	1.981	2.268
$(GT)_{15}$	Dox	(9,4)	3.166	0.247
$(GT)_{15}$	Dox	(9,5+10,3)	2.314	1.616
$(GT)_{15}$	Epi	(10,2)	1.906	Fit failed
$(GT)_{15}$	Epi	(7,5)	2.027	Fit failed
$(GT)_{15}$	Epi	(7,6)	1.937	Fit failed
$(GT)_{15}$	Epi	(8,6)	2.25	Fit failed
$(GT)_{15}$	Epi	(8,7)	1.256	108
$(GT)_{15}$	Epi	(9,4)	1.416	Fit failed
$(GT)_{15}$	Epi	(9,5+10,3)	194.9	Fit failed
$(GT)_{15}$	Ida	(10,2)	6.619	Fit failed
$(GT)_{15}$	Ida	(7,5)	6.871	Fit failed
$(GT)_{15}$	Ida	(7,6)	6.631	Fit failed
$(GT)_{15}$	Ida	(8,6)	2.721	Fit failed
$(GT)_{15}$	Ida	(8,7)	2.071	Fit failed

$(GT)_{15}$	Ida	(9,4)	4.249	Fit failed
$(GT)_{15}$	Ida	(9,5+10,3)	Fit failed	Fit failed
TTA TAT TAT ATT	Dauno	(10,2)	2.932	Fit failed
TTA TAT TAT ATT	Dauno	(7,5)	3.349	Fit failed
TTA TAT TAT ATT	Dauno	(7,6)	3.524	0.75
TTA TAT TAT ATT	Dauno	(8,6)	3.911	Fit failed
TTA TAT TAT ATT	Dauno	(8,7)	1.664	Fit failed
TTA TAT TAT ATT	Dauno	(9,4)	4.047	Fit failed
TTA TAT TAT ATT	Dauno	(9,5+10,3)	1.643	Fit failed
TTA TAT TAT ATT	Dox	(10,2)	5.11	1.303
TTA TAT TAT ATT	Dox	(7,5)	4.914	1.53
TTA TAT TAT ATT	Dox	(7,6)	4.223	1.255
TTA TAT TAT ATT	Dox	(8,6)	8.85	0.93
TTA TAT TAT ATT	Dox	(8,7)	6.372	1.266
TTA TAT TAT ATT	Dox	(9,4)	6.245	0.727
TTA TAT TAT ATT	Dox	(9,5+10,3)	3.795	1.174
TTA TAT TAT ATT	Epi	(10,2)	5.11	0.863
TTA TAT TAT ATT	Epi	(7,5)	3.945	Fit failed
TTA TAT TAT ATT	Epi	(7,6)	2.512	0.738
TTA TAT TAT ATT	Epi	(8,6)	4.483	Fit failed
TTA TAT TAT ATT	Epi	(8,7)	3.307	Fit failed
TTA TAT TAT ATT	Epi	(9,4)	3.33	0.277
TTA TAT TAT ATT	Epi	(9,5+10,3)	7.184	0.507
TTA TAT TAT ATT	Ida	(10,2)	3.748	1.278
TTA TAT TAT ATT	Ida	(7,5)	9.713	1.02

TTA TAT TAT ATT	Ida	(7,6)	5.254	0.981
TTA TAT TAT ATT	Ida	(8,6)	5.074	Fit failed
TTA TAT TAT ATT	Ida	(8,7)	9.396	0.796
TTA TAT TAT ATT	Ida	(9,4)	9.605	Fit failed
TTA TAT TAT ATT	Ida	(9,5+10,3)	5.672	Fit failed
$(TCG)_4TC$	Dauno	(10,2)	18.47	Fit failed
$(TCG)_4TC$	Dauno	(7,5)	29.48	0.834
$(TCG)_4TC$	Dauno	(7,6)	9.18	Fit failed
$(TCG)_4TC$	Dauno	(8,6)	12.21	Fit failed
$(TCG)_4TC$	Dauno	(8,7)	Fit failed	Fit failed
$(TCG)_4TC$	Dauno	(9,4)	10.2	Fit failed
$(TCG)_4TC$	Dauno	(9,5+10,3)	Fit failed	Fit failed
$(TCG)_4TC$	Dox	(10,2)	60.9	0.649
$(TCG)_4TC$	Dox	(7,5)	60.61	2.82
$(TCG)_4TC$	Dox	(7,6)	64.27	1.1
$(TCG)_4TC$	Dox	(8,6)	32.17	1.322
$(TCG)_4TC$	Dox	(8,7)	Fit failed	2.079
$(TCG)_4TC$	Dox	(9,4)	32.89	0.562
$(TCG)_4TC$	Dox	(9,5+10,3)	Fit failed	1.394
$(TCG)_4TC$	Epi	(10,2)	10.66	Fit failed
$(TCG)_4TC$	Epi	(7,5)	8.839	Fit failed
$(TCG)_4TC$	Epi	(7,6)	Fit failed	Fit failed
$(TCG)_4TC$	Epi	(8,6)	11.43	1.021
$(TCG)_4TC$	Epi	(8,7)	Fit failed	Fit failed
$(TCG)_4TC$	Epi	(9,4)	9.134	Fit failed
$(TCG)_4TC$	Epi	(9,5+10,3)	2.141	Fit failed
$(TCG)_4TC$	Ida	(10,2)	Fit failed	Fit failed

$(TCG)_4TC$	Ida	(7,5)	19.24	Fit failed
$(TCG)_4TC$	Ida	(7,6)	12.65	Fit failed
$(TCG)_4TC$	Ida	(8,6)	15.2	Fit failed
$(TCG)_4TC$	Ida	(8,7)	10.64	Fit failed
$(TCG)_4TC$	Ida	(9,4)	8.752	Fit failed
$((TCG)_4TC$	Ida	(9,5+10,3)	7.325	Fit failed
CCG CGG	Dauno	(10,2)	Fit failed	Fit failed
CCG CGG	Dauno	(7,5)	34.76	Fit failed
CCG CGG	Dauno	(7,6)	6.803	Fit failed
CCG CGG	Dauno	(8,6)	3.536	Fit failed
CCG CGG	Dauno	(8,7)	Fit failed	Fit failed
CCG CGG	Dauno	(9,4)	9.385	Fit failed
CCG CGG	Dauno	(9,5+10,3)	Fit failed	Fit failed
CCG CGG	Dox	(10,2)	Fit failed	Fit failed
CCG CGG	Dox	(7,5)	100.1	1.599
CCG CGG	Dox	(7,6)	8.531	0.75
CCG CGG	Dox	(8,6)	Fit failed	Fit failed
CCG CGG	Dox	(8,7)	2.179	1.345
CCG CGG	Dox	(9,4)	9.128	0.565
CCG CGG	Dox	(9,5+10,3)	Fit failed	1.323
CCG CGG	Epi	(10,2)	Fit failed	Fit failed
CCG CGG	Epi	(7,5)	6.334	Fit failed
CCG CGG	Epi	(7,6)	2.96	0.465
CCG CGG	Epi	(8,6)	Fit failed	Fit failed
CCG CGG	Epi	(8,7)	Fit failed	1.042
CCG CGG	Epi	(9,4)	3.605	0.304
CCG CGG	Epi	(9,5+10,3)	Fit failed	1.048

CCG CGG	Ida	(10,2)	Fit failed	Fit failed
CCG CGG	Ida	(7,5)	14.59	Fit failed
CCG CGG	Ida	(7,6)	3.41	Fit failed
CCG CGG	Ida	(8,6)	3.799	Fit failed
CCG CGG	Ida	(8,7)	0.616	Fit failed
CCG CGG	Ida	(9,4)	5.148	Fit failed
CCG CGG	Ida	(9,5+10,3)	Fit failed	Fit failed
$(ATTT)_4$	Dauno	(10,2)	7.575	Fit failed
$(ATTT)_4$	Dauno	(7,5)	8.831	2.214
$(ATTT)_4$	Dauno	(7,6)	5.382	1.63
$(ATTT)_4$	Dauno	(8,6)	Fit failed	Fit failed
$(ATTT)_4$	Dauno	(8,7)	Fit failed	Fit failed
$(ATTT)_4$	Dauno	(9,4)	4.91	1.586
$(ATTT)_4$	Dauno	(9,5+10,3)	2.635	Fit failed
$(ATTT)_4$	Dox	(10,2)	8.646	0.134
$(ATTT)_4$	Dox	(7,5)	20.49	3.393
$(ATTT)_4$	Dox	(7,6)	Fit failed	1.589
$(ATTT)_4$	Dox	(8,6)	Fit failed	Fit failed
$(ATTT)_4$	Dox	(8,7)	Fit failed	Fit failed
$(ATTT)_4$	Dox	(9,4)	Fit failed	1.523
$(ATTT)_4$	Dox	(9,5+10,3)	Fit failed	1.582
$(ATTT)_4$	Epi	(10,2)	1.948	Fit failed
$(ATTT)_4$	Epi	(7,5)	Fit failed	Fit failed
$(ATTT)_4$	Epi	(7,6)	Fit failed	0.967
$(ATTT)_4$	Epi	(8,6)	Fit failed	Fit failed
$(ATTT)_4$	Epi	(8,7)	Fit failed	Fit failed
$(ATTT)_4$	Epi	(9,4)	Fit failed	0.965

$(ATTT)_4$	Epi	(9,5+10,3)	1.544	Fit failed
$(ATTT)_4$	Ida	(10,2)	9.144	Fit failed
$(ATTT)_4$	Ida	(7,5)	11.95	3.628
$(ATTT)_4$	Ida	(7,6)	7.153	1.45
$(ATTT)_4$	Ida	(8,6)	Fit failed	Fit failed
$(ATTT)_4$	Ida	(8,7)	Fit failed	Fit failed
$(ATTT)_4$	Ida	(9,4)	10.45	Fit failed
$(ATTT)_4$	Ida	(9,5+10,3)	1.648	Fit failed
GGA CGC	Dauno	(10,2)	20.14	1.611
GGA CGC	Dauno	(7,5)	22.88	2.631
GGA CGC	Dauno	(7,6)	5.576	1.517
GGA CGC	Dauno	(8,6)	Fit failed	Fit failed
GGA CGC	Dauno	(8,7)	Fit failed	0.181
GGA CGC	Dauno	(9,4)	13.14	2.274
GGA CGC	Dauno	(9,5+10,3)	13.37	Fit failed
GGA CGC	Dox	(10,2)	Fit failed	3.474
GGA CGC	Dox	(7,5)	13.72	4.133
GGA CGC	Dox	(7,6)	3.527	2.145
GGA CGC	Dox	(8,6)	Fit failed	Fit failed
GGA CGC	Dox	(8,7)	15.15	0.691
GGA CGC	Dox	(9,4)	6.171	2.202
GGA CGC	Dox	(9,5+10,3)	42.21	4.198
GGA CGC	Epi	(10,2)	37.84	1.724
GGA CGC	Epi	(7,5)	Fit failed	2.715
GGA CGC	Epi	(7,6)	Fit failed	1.836
GGA CGC	Epi	(8,6)	Fit failed	Fit failed
GGA CGC	Epi	(8,7)	1.586	0.687
GGA CGC	Epi	(9,4)	2.634	1.379
GGA CGC	Epi	(9,5+10,3)	18.51	0.712
GGA CGC	Ida	(10,2)	69.76	1.617
GGA CGC	Ida	(7,5)	29.28	3.251
GGA CGC	Ida	(7,6)	6.121	1.531

GGA CGC	Ida	(8,6)	Fit failed	Fit failed
GGA CGC	Ida	(8,7)	1.69	Fit failed
GGA CGC	Ida	(9,4)	4.328	1.107
GGA CGC	Ida	(9,5+10,3)	3.888	0.791
$(TAT)_6$	Dauno	(10,2)	2.221	Fit failed
$(TAT)_6$	Dauno	(7,5)	2.417	Fit failed
$(TAT)_6$	Dauno	(7,6)	3.206	Fit failed
$(TAT)_6$	Dauno	(8,6)	3.594	Fit failed
$(TAT)_6$	Dauno	(8,7)	1.699	Fit failed
$(TAT)_6$	Dauno	(9,4)	1.7	Fit failed
$(TAT)_6$	Dauno	(9,5+10,3)	Fit failed	3.92
$(TAT)_6$	Dox	(10,2)	5.731	Fit failed
$(TAT)_6$	Dox	(7,5)	4.86	0.307
$(TAT)_6$	Dox	(7,6)	5.594	0.734
$(TAT)_6$	Dox	(8,6)	4.463	Fit failed
$(TAT)_6$	Dox	(8,7)	2.369	Fit failed
$(TAT)_6$	Dox	(9,4)	3.922	Fit failed
$(TAT)_6$	Dox	(9,5+10,3)	Fit failed	Fit failed
$(TAT)_6$	Epi	(10,2)	3.088	Fit failed
$(TAT)_6$	Epi	(7,5)	2.751	Fit failed
$(TAT)_6$	Epi	(7,6)	4.219	Fit failed
$(TAT)_6$	Epi	(8,6)	3.165	Fit failed
$(TAT)_6$	Epi	(8,7)	1.956	Fit failed
$(TAT)_6$	Epi	(9,4)	1.969	Fit failed
$(TAT)_6$	Epi	(9,5+10,3)	Fit failed	Fit failed
$(TAT)_6$	Ida	(10,2)	9.941	Fit failed

$(TAT)_6$	Ida	(7,5)	5.397	Fit failed
$(TAT)_6$	Ida	(7,6)	5.196	Fit failed
$(TAT)_6$	Ida	(8,6)	4.417	Fit failed
$(TAT)_6$	Ida	(8,7)	Fit failed	Fit failed
$(TAT)_6$	Ida	(9,4)	3.738	Fit failed
$(TAT)_6$	Ida	(9,5+10,3)	Fit failed	4.813
$(GTT)_3G$	Dauno	(10,2)	4.39	Fit failed
$(GTT)_3G$	Dauno	(7,5)	5.759	Fit failed
$(GTT)_3G$	Dauno	(7,6)	3.72	Fit failed
$(GTT)_3G$	Dauno	(8,6)	3.895	Fit failed
$(GTT)_3G$	Dauno	(8,7)	Fit failed	Fit failed
$(GTT)_3G$	Dauno	(9,4)	4.065	27.99
$(GTT)_3G$	Dauno	(9,5+10,3)	Fit failed	22.58
$(GTT)_3G$	Dox	(10,2)	4.682	Fit failed
$(GTT)_3G$	Dox	(7,5)	4.913	Fit failed
$(GTT)_3G$	Dox	(7,6)	5.042	Fit failed
$(GTT)_3G$	Dox	(8,6)	4.234	Fit failed
$(GTT)_3G$	Dox	(8,7)	Fit failed	Fit failed
$(GTT)_3G$	Dox	(9,4)	5.03	Fit failed
$(GTT)_3G$	Dox	(9,5+10,3)	Fit failed	Fit failed
$(GTT)_3G$	Epi	(10,2)	2.175	Fit failed
$(GTT)_3G$	Epi	(7,5)	1.559	Fit failed
$(GTT)_3G$	Epi	(7,6)	Fit failed	Fit failed
$(GTT)_3G$	Epi	(8,6)	1.83	Fit failed
$(GTT)_3G$	Epi	(8,7)	Fit failed	Fit failed

$(GTT)_3G$	Epi	(9,4)	2.831	Fit failed
$(GTT)_3G$	Epi	(9,5+10,3)	Fit failed	Fit failed
$(GTT)_3G$	Ida	(10,2)	3.075	Fit failed
$(GTT)_3G$	Ida	(7,5)	3.547	Fit failed
$(GTT)_3G$	Ida	(7,6)	2.542	Fit failed
$(GTT)_3G$	Ida	(8,6)	2.708	Fit failed
$(GTT)_3G$	Ida	(8,7)	Fit failed	Fit failed
$(GTT)_3G$	Ida	(9,4)	3.109	Fit failed
$(GTT)_3G$	Ida	(9,5+10,3)	6.145	Fit failed
$(GT)_6$	Dauno	(10,2)	11.09	0.452
$(GT)_6$	Dauno	(7,5)	6.947	Fit failed
$(GT)_6$	Dauno	(7,6)	5.26	Fit failed
$(GT)_6$	Dauno	(8,6)	9.435	Fit failed
$(GT)_6$	Dauno	(8,7)	2.386	40.04
$(GT)_6$	Dauno	(9,4)	4.707	Fit failed
$(GT)_6$	Dauno	(9,5+10,3)	Fit failed	Fit failed
$(GT)_6$	Dox	(10,2)	Fit failed	0.958
$(GT)_6$	Dox	(7,5)	8.944	0.761
$(GT)_6$	Dox	(7,6)	3.967	0.752
$(GT)_6$	Dox	(8,6)	14.6	Fit failed
$(GT)_6$	Dox	(8,7)	Fit failed	Fit failed
$(GT)_6$	Dox	(9,4)	4.934	0.396
$(GT)_6$	Dox	(9,5+10,3)	3.293	1.954
$(GT)_6$	Epi	(10,2)	Fit failed	Fit failed
$(GT)_6$	Epi	(7,5)	3.296	Fit failed
$(GT)_6$	Epi	(7,6)	Fit failed	Fit failed
$(GT)_6$	Epi	(8,6)	2.806	Fit failed

$(GT)_6$	Epi	(8,7)	Fit failed	Fit failed
$(GT)_6$	Epi	(9,4)	2.036	Fit failed
$(GT)_6$	Epi	(9,5+10,3)	2.838	Fit failed
$(GT)_6$	Ida	(10,2)	74.69	Fit failed
$(GT)_6$	Ida	(7,5)	Fit failed	Fit failed
$(GT)_6$	Ida	(7,6)	4.629	Fit failed
$(GT)_6$	Ida	(8,6)	9.828	Fit failed
$(GT)_6$	Ida	(8,7)	3.317	Fit failed
$(GT)_6$	Ida	(9,4)	4.741	Fit failed
$(GT)_6$	Ida	(9,5+10,3)	Fit failed	Fit failed
$(CTG)_3C$	Dauno	(10,2)	53.73	0.426
$(CTG)_3C$	Dauno	(7,5)	60.34	0.809
$(CTG)_3C$	Dauno	(7,6)	39.24	0.207
$(CTG)_3C$	Dauno	(8,6)	37.25	Fit failed
$(CTG)_3C$	Dauno	(8,7)	Fit failed	Fit failed
$(CTG)_3C$	Dauno	(9,4)	31.47	Fit failed
$(CTG)_3C$	Dauno	(9,5+10,3)	2.571	Fit failed
$(CTG)_3C$	Dox	(10,2)	64.13	1.198
$(CTG)_3C$	Dox	(7,5)	40.21	2.743
$(CTG)_3C$	Dox	(7,6)	43.88	0.833
$(CTG)_3C$	Dox	(8,6)	25.2	0.62
$(CTG)_3C$	Dox	(8,7)	Fit failed	2.154
$(CTG)_3C$	Dox	(9,4)	49.21	0.634
$(CTG)_3C$	Dox	(9,5+10,3)	2.576	1.104
$(CTG)_3C$	Epi	(10,2)	11.41	0.633
$(CTG)_3C$	Epi	(7,5)	7.849	3.458
$(CTG)_3C$	Epi	(7,6)	14.43	0.693
$(CTG)_3C$	Epi	(8,6)	13.16	0.318
$(CTG)_3C$	Epi	(8,7)	Fit failed	1.829
$(CTG)_3C$	Epi	(9,4)	7.53	0.262

$(CTG)_3C$	Epi	(9,5+10,3)	Fit failed	1.114
$(CTG)_3C$	Ida	(10,2)	27.58	Fit failed
$(CTG)_3C$	Ida	(7,5)	50.46	Fit failed
$(CTG)_3C$	Ida	(7,6)	22.63	Fit failed
$(CTG)_3C$	Ida	(8,6)	27.6	Fit failed
$(CTG)_3C$	Ida	(8,7)	Fit failed	Fit failed
$(CTG)_3C$	Ida	(9,4)	10.35	Fit failed
$(CTG)_3C$	Ida	(9,5+10,3)	12.55	Fit failed
$(T)_{12}$	Dauno	(10,2)	2.577	Fit failed
$(T)_{12}$	Dauno	(7,5)	1.928	Fit failed
$(T)_{12}$	Dauno	(7,6)	2.27	Fit failed
$(T)_{12}$	Dauno	(8,6)	1.847	5.854
$(T)_{12}$	Dauno	(8,7)	1.138	Fit failed
$(T)_{12}$	Dauno	(9,4)	1.975	Fit failed
$(T)_{12}$	Dauno	(9,5+10,3)	0.302	Fit failed
$(T)_{12}$	Dox	(10,2)	3.714	Fit failed
$(T)_{12}$	Dox	(7,5)	3.982	Fit failed
$(T)_{12}$	Dox	(7,6)	2.971	Fit failed
$(T)_{12}$	Dox	(8,6)	2.473	Fit failed
$(T)_{12}$	Dox	(8,7)	1.596	Fit failed
$(T)_{12}$	Dox	(9,4)	3.576	12.16
$(T)_{12}$	Dox	(9,5+10,3)	Fit failed	Fit failed
$(T)_{12}$	Epi	(10,2)	1.657	Fit failed
$(T)_{12}$	Epi	(7,5)	0.999	Fit failed
$(T)_{12}$	Epi	(7,6)	1.434	Fit failed

$(T)_{12}$	Epi	(8,6)	1.197	Fit failed
$(T)_{12}$	Epi	(8,7)	0.856	4.518
$(T)_{12}$	Epi	(9,4)	1.259	Fit failed
$(T)_{12}$	Epi	(9,5+10,3)	Fit failed	Fit failed
$(T)_{12}$	Ida	(10,2)	3.097	Fit failed
$(T)_{12}$	Ida	(7,5)	1.944	Fit failed
$(T)_{12}$	Ida	(7,6)	2.351	Fit failed
$(T)_{12}$	Ida	(8,6)	2.848	Fit failed
$(T)_{12}$	Ida	(8,7)	1.49	Fit failed
$(T)_{12}$	Ida	(9,4)	2.571	Fit failed
$(T)_{12}$	Ida	(9,5+10,3)	Fit failed	Fit failed
$(GT)_{30}$	Dauno	(10,2)	8.105	0.217
$(GT)_{30}$	Dauno	(7,5)	9.52	0.291
$(GT)_{30}$	Dauno	(7,6)	9.743	0.226
$(GT)_{30}$	Dauno	(8,6)	5.318	Fit failed
$(GT)_{30}$	Dauno	(8,7)	3.174	Fit failed
$(GT)_{30}$	Dauno	(9,4)	4.968	Fit failed
$(GT)_{30}$	Dauno	(9,5+10,3)	Fit failed	Fit failed
$(GT)_{30}$	Dox	(10,2)	Fit failed	0.581
$(GT)_{30}$	Dox	(7,5)	11.07	1.34
$(GT)_{30}$	Dox	(7,6)	Fit failed	0.886
$(GT)_{30}$	Dox	(8,6)	9.915	0.285
$(GT)_{30}$	Dox	(8,7)	4.676	2.959
$(GT)_{30}$	Dox	(9,4)	3.331	0.239
$(GT)_{30}$	Dox	(9,5+10,3)	3.69	1.535
$(GT)_{30}$	Epi	(10,2)	Fit failed	Fit failed
$(GT)_{30}$	Epi	(7,5)	2.066	Fit failed
$(GT)_{30}$	Epi	(7,6)	Fit failed	Fit failed

$(GT)_{30}$	Epi	(8,6)	3.532	Fit failed
$(GT)_{30}$	Epi	(8,7)	2.776	Fit failed
$(GT)_{30}$	Epi	(9,4)	1.216	Fit failed
$(GT)_{30}$	Epi	(9,5+10,3)	42.5	Fit failed
$(GT)_{30}$	Ida	(10,2)	8.464	Fit failed
$(GT)_{30}$	Ida	(7,5)	10.49	Fit failed
$(GT)_{30}$	Ida	(7,6)	9.884	Fit failed
$(GT)_{30}$	Ida	(8,6)	3.695	Fit failed
$(GT)_{30}$	Ida	(8,7)	3.307	Fit failed
$(GT)_{30}$	Ida	(9,4)	5.324	Fit failed
$(GT)_{30}$	Ida	(9,5+10,3)	Fit failed	Fit failed

Supplementary Table 3. Performance of SVM models for binary concentration classification in buffer and biological matrices in PCA plot. (CV: cross-validation for training; Validation: synthetic urine and sweat)

	**CV accuracy**	**Test accuracy**	**Validation accuracy**
**Daunorubicin**	1.0000 ± 0.0000	1.0000	1.0000
**Doxorubicin**	1.0000 ± 0.0000	0.5000	0.5000
**Epirubicin**	1.0000 ± 0.0000	0.5000	0.5000
**Idarubicin**	1.0000 ± 0.0000	1.0000	1.0000

Supplementary Table 4. Top 10 ssDNA-(n,m) combinations with highest absolute PC1 loadings for each anthracycline.

	**Feature**	**PC1**	**PC2**	**PC3**
**Daunorubicin**	$(TAT)_6$*(10,2)_*wl*	0.851983	-0.07454	-0.17132
	$(TAT)_6$*(9,4)_*wl*	0.382835	-0.06947	0.53657

	(TAT)$_6$*(7,5)_*wl*	0.143268	0.003955	0.051422
	(TAT)$_6$*(9,4)_*int*	0.11432	0.488973	-0.24496
	(T)$_{12}$*(9,4)_*wl*	0.112942	-0.15777	-0.52361
	(GT)$_{15}$*(8,7)_*wl*	0.106332	-0.08085	-0.0621
	GGACGC*(7,6)_*int*	-0.09681	-0.07074	0.010415
	TTATATTATATT*(9,5)+(10,3)_*wl*	0.094292	-0.07513	-0.18019
	TTATATTATATT*(7,6)_*int*	0.067505	0.314878	-0.09441
	(GT)$_{15}$*(8,6)_*wl*	0.058032	0.034321	-0.00064
**Doxorubicin**	(T)$_{12}$*(9,4)_*int*	0.901616	-0.13951	0.067469
	(T)$_{12}$*(7,6)_*int*	0.30983	-0.08299	0.063758
	(T)$_{12}$*(7,5)_*int*	0.172457	-0.07544	0.045074
	CCGCGG*(9,5)+(10,3)_*int*	-0.07134	-0.24448	0.294141
	(T)$_{12}$*(7,5)_*wl*	0.069365	0.102523	-0.15449
	(GTT)$_3$G*(7,5)_*wl*	0.066429	0.126433	-0.0506
	(TCG)$_4$TC*(9,5)+(10,3)_*wl*	0.053682	0.063033	0.027989
	(TCG)$_4$TC*(7,6)_*wl*	-0.04795	-0.17822	0.223415
	GGACGC*(7,6)_*int*	-0.0443	-0.23969	0.153528

	$(T)_{12}$*(7,6)_*wl*	0.042971	0.015118	-0.06474
**Epirubicin**	$(TAT)_6$*(7,6)_*int*	0.408366	0.090613	-0.07485
	$(GTT)_3G$*(9,5)+(10,3)_*int*	0.380723	0.080929	-0.10661
	$(TAT)_6$*(9,4)_*int*	0.309901	0.049348	-0.05938
	TTATATTATATT*(7,5)_*int*	0.250937	0.09675	0.046434
	$(GTT)_3G$*(7,6)_*int*	0.196542	0.018519	-0.01875
	TTATATTATATT*(9,4)_*int*	0.193264	0.094664	0.101211
	$(TCG)_4TC$*(9,4)_*int*	0.187575	0.098859	-0.13959
	$(GTT)_3G$*(9,4)_*int*	0.162952	0.002705	0.007055
	$(GT)_{15}$*(7,6)_*int*	0.15612	0.004774	-0.05593
	$(GT)_{15}$*(9,4)_*int*	0.149052	0.002878	-0.04324
**Idarubicin**	$(GT)_{30}$*(9,4)_*int*	0.315611	0.068914	-0.17637
	CCGCGG*(9,5)+(10,3)_*int*	0.299782	0.130549	-0.16999
	$(GT)_{30}$*(7,6)_*int*	0.282599	0.162562	-0.18965
	$(GTT)_3G$*(7,6)_*int*	0.265926	0.02377	-0.14202
	$(GTT)_3G$*(9,4)_*int*	0.251676	0.00191	-0.16277
	$(GT)_{30}$*(7,5)_*int*	0.208016	0.135192	-0.10672

	$(GT)_6$*(7,5)_*int*	0.16618	-0.0013	-0.02796
	$(GT)_{30}$*(10,2)_*int*	0.15978	0.109552	-0.0733
	CCGCGG*(7,6)_*int*	0.151966	0.119974	-0.03878
	$(GT)_{15}$*(7,6)_*int*	0.151453	0.107696	-0.02382

Supplemental References

(1) Cohen, Z.; Parveen, S.; Williams, R. M. Optimization of Ssdna-Swcnt Ultracentrifugation Via Efficacy Measurements. *ECS Journal of Solid State Science and Technology* **2022**, *11* (10).

(2) Ryan, A. K.; Rahman, S.; Williams, R. M. Optical Aptamer-Based Cytokine Nanosensor Detects Macrophage Activation by Bacterial Toxins. *ACS Sensors* **2024**, *9* (7), 3697-3706.

(3) Gaikwad, P.; Rahman, N.; Parikh, R.; Crespo, J.; Cohen, Z.; Williams, R. M. Optical Nanosensor Passivation Enables Highly Sensitive Detection of the Inflammatory Cytokine Interleukin-6. *ACS Applied Materials & Interfaces* **2024**, *16* (21), 27102-27113.

(4) Cohen, Z.; Alpert, D. J.; Weisel, A. C.; Ryan, A.; Roach, A.; Rahman, S.; Gaikwad, P. V.; Nicoll, S. B.; Williams, R. M. Noninvasive Injectable Optical Nanosensor-Hydrogel Hybrids Detect Doxorubicin in Living Mice. *Advanced optical materials* **2024**, *12* (17), 2303324.

(5) Del Bonis-O'Donnell, J. T.; Pinals, R. L.; Jeong, S.; Thakrar, A.; Wolfinger, R. D.; Landry, M. P. Chemometric Approaches for Developing Infrared Nanosensors to Image Anthracyclines. *Biochemistry* **2019**, *58* (1), 54-64.

(6) Pedregosa, F.; Varoquaux, G.; Gramfort, A.; Michel, V.; Thirion, B.; Grisel, O.; Blondel, M.; Prettenhofer, P.; Weiss, R.; Dubourg, V. Scikit-Learn: Machine Learning in Python. *the Journal of machine Learning research* **2011**, *12*, 2825-2830.

(7) Lundberg, S. M.; Lee, S.-I. A Unified Approach to Interpreting Model Predictions. *Advances in neural information processing systems* **2017**, *30*.
